# Bending Modulus of Lipid Membranes from Density Correlation Functions

**DOI:** 10.1021/acs.jctc.2c00099

**Published:** 2022-04-07

**Authors:** Jose Hernández-Muñoz, Fernando Bresme, Pedro Tarazona, Enrique Chacón

**Affiliations:** † Departamento de Física Teórica de la Materia Condensada, IFIMAC Condensed Matter Physics Center, Universidad Autónoma de Madrid, Madrid 28049, Spain; ‡ Department of Chemistry, Molecular Sciences Research Hub, Imperial College, W12 0BZ, London, United Kingdom; ¶ Instituto Nicolás Cabrera de Ciencia de Materiales, Universidad Autónoma de Madrid, Madrid 28049, Spain; ∥ Instituto de Ciencia de Materiales de Madrid, Consejo Superior de Investigaciones Científicas, Madrid 28049, Spain

## Abstract

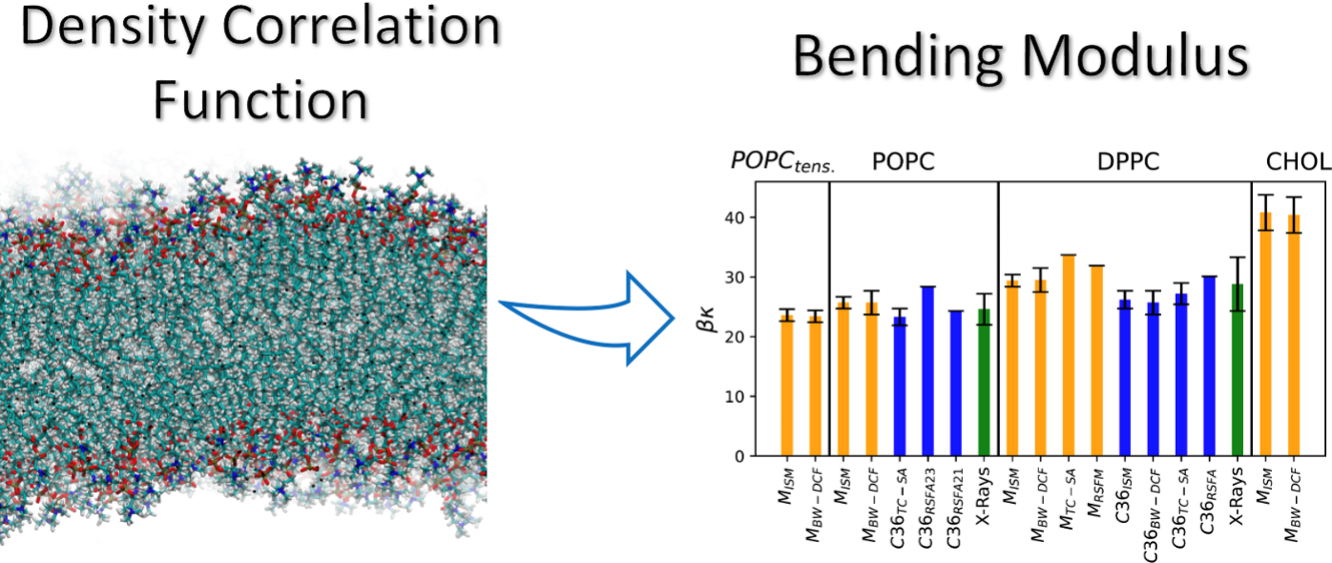

The bending modulus κ quantifies the elasticity of biological membranes in terms of the free energy cost of increasing the membrane corrugation. Molecular dynamics (MD) simulations provide a powerful approach to quantify κ by analyzing the thermal fluctuations of the lipid bilayer. However, existing methods require the identification and filtering of non-mesoscopic fluctuation modes. State of the art methods rely on identifying a smooth surface to describe the membrane shape. These methods introduce uncertainties in calculating κ since they rely on different criteria to select the relevant fluctuation modes. Here, we present a method to compute κ using molecular simulations. Our approach circumvents the need to define a mesoscopic surface or an orientation field for the lipid tails explicitly. The bending and tilt moduli can be extracted from the analysis of the density correlation function (DCF). The method introduced here builds on the Bedeaux and Weeks (BW) theory for the DCF of fluctuating interfaces and on the coupled undulatory (CU) mode introduced by us in previous work. We test the BW-DCF method by computing the elastic properties of lipid membranes with different system sizes (from 500 to 6000 lipid molecules) and using coarse-grained (for POPC and DPPC lipids) and fully atomistic models (for DPPC). Further, we quantify the impact of cholesterol on the bending modulus of DPPC bilayers. We compare our results with bending moduli obtained with X-ray diffraction data and different computer simulation methods.

## 1 Introduction

Lipids self-assemble spontaneously in water forming bilayers with the lipid hydrophobic tails shielded from water by the polar head groups. These soft-matter pseudo-2D structures provide a robust physical barrier for the cell^1,2^ and host membrane proteins that enable many biochemical processes. The lipid membrane thickness varies with lipid composition, and it is typically *d* ≈ 4–5 nm. At length scales much larger than the membrane thickness the Helfrich Hamiltonian^3^ (HH) defines the elastic (free) energy in terms of the geometrical characteristics of a mathematical surface defining the membrane shape. The HH can be used to analyze the equilibrium shape of large lipid assemblies, such as lipid vesicles, vesicle deformation under experimental conditions (e.g., using aspiration micropipettes method^4,5^), and the thermal fluctuations of free and supported bilayers.^6−9^

The surface tension, γ_o_ (times the area), and the bending modulus κ (times the mean square curvature) are the key terms defining the HH. Unlike the surface tension, the bending modulus κ cannot be modified externally, but it depends on the composition of the membrane and the temperature.^10^ The values of γ_o_ and κ relate the equilibrium shape of the membrane and its fluctuations to the composition of the lipids and the experimental conditions (temperature and tensile stress). Helfrich’s theory has been expanded to incorporate a spontaneous curvature term to account for asymmetry in lipid membranes^11^ that may appear in multicomponent mixtures, such as in the cell membrane. In vitro symmetric membranes^12−14^ have zero spontaneous curvature. Other terms, such as the Gaussian characteristics and the edge of a membrane can be added to the HH, but in general, such terms are not needed to investigate membranes under typical experimental conditions. Simulated (in silico) symmetric membranes^15−19^ are often studied using the HH of a fluctuating surface, *z* = ξ(*x⃗*) = ∑_*q⃗*_ ξ̂_*q⃗*_*e*^*iq⃗x⃗*^, with *x⃗* ≡ (*x*, *y*), and wavevectors *q⃗* ≡ (*q*_*x*_, *q*_*y*_) with values determined by the lateral length of the simulation box, *L*, and the periodic boundary conditions. The Fourier amplitudes ξ̂_*q⃗*_ fluctuate following Gaussian probability distributions with mean square values ⟨|ξ̂_*q⃗*_|^2^⟩, and mean equal to zero.

The calculation of κ from molecular dynamics (MD) simulations is far from trivial, and several methods have been developed over the past decade. The most popular methods rely on the spectral analysis of the thermal fluctuations of the membrane shape and the orientation of the aliphatic tails.^20−22^ Erguder and Deserno^23^ have reviewed the challenges associated with the computation of κ. First, a smooth mathematical surface *z* = ξ(*x⃗*) (which requires a lattice representation describing the membrane shape) and a smooth local field *n⃗*(*x⃗*) (for the tilt of the lipid molecules) must be defined using the atomic coordinates of the lipid molecules. The approaches followed to construct that surface, the local tilt field, and the fluctuation analysis that links ξ(*x⃗*) and *n⃗*(*x⃗*), are not unique. As a matter of fact, there are several methods^10,23−25^ to perform tilt-curvature spectral analysis (TC-SA). The differences in the methodological approaches introduce uncertainties regarding the choice of parameters and the criteria required to construct the smooth surfaces. Specifically, one problem is concerned with the choice of upper bound, *q*, in the Fourier transform of the surface, ξ̂_*q⃗*_, that is defined over a regular grid, that is, disregarding the off-grid molecular coordinates.^23^ A method was proposed^26^ to circumvent these problems, using a direct Fourier transform (DFT) of the lipid positions instead, hence avoiding the computation of a continuous smooth surface. However, the high-*q* lateral correlations can only be approximately subtracted, and this shortcoming is reflected in the calculation of the bending modulus.^27^ The direct least-squares fitting of the lipid positions,^28^ as an alternative to the DFT description has the same problems as the regular-grid Fourier transform TC-SA, namely, the choice of an upper bound for *q*.

Moreover, the direct use of the HH to evaluate the bending modulus entails some problems, concerned with the practical limitations in the simulation system size and time. Helfrich’s description applies to membrane fluctuations with wavelengths much longer than the membrane thickness. Therefore, an accurate evaluation of κ requires huge systems, and extremely long MD trajectories, to sample the slow low-*q* modes. Hence, the computation is impractical in many cases, particularly for all-atoms force-fields. Therefore, the community has devised alternative approaches to compute κ, which rely on extensions of the theory to shorter length scales (higher *q*), by including additional elastic modes that go beyond the overall undulation of the membrane considered in the HH. Hence, fitting the fluctuation spectrum over a wide *q* range requires new elastic constants, with κ being one of the fitting parameters. These approaches have been used to estimate κ using minimal systems (a few hundred and fewer lipids) and short MD simulations. In our opinion, the bending modulus obtained through this approach depends on the criterion used to analyze the membrane fluctuations, hence bringing uncertainty to the computations.

As an alternative to the TC-SA methods, the membrane shape has also been modeled using two smooth mesoscopic surfaces *z* = ξ^±^(*x⃗*) for the “upper” (+) and “lower” (−) membrane layers without targetting the tail orientations. In this approach, the undulatory (U) and peristaltic (P) eigenmodes in the correlation matrix for the two surfaces^29^ provide a natural choice to define long and short wavelength fluctuations, describing either collective or individual motions of lipids, respectively. The Helfrich Hamiltonian describes the fluctuations of the U mode via the mean surface *z* = ξ^U^(*x⃗*) = (ξ^+^(*x⃗*) + ξ^–^(*x⃗*))/2. However, a gradual decoupling of the fluctuations at the two monolayers that form the membrane takes place at short length-scales. Therefore, the mesoscopic fluctuations of ξ^U^(*x⃗*) and the peristaltic fluctuations in the local membrane thickness, ξ^+^(*x⃗*)−ξ^–^(*x⃗*), mix with each other. The molecular protrusions dominate the fluctuations of the lipid monolayers (m) at high-*q*, when each monolayer fluctuates nearly independently of the other. We proposed^19^ an approach to describe that decoupling of the fluctuations of the two monolayers, by introducing a coupled-undulatory (CU) mode. Because the length of the lipid tails and their tilt^28^ are correlated, the description of the membrane elasticity in terms of U, P, and CU fluctuation modes and the curvature-tilt analysis are linked. Therefore, we may expect that some elastic constants beyond the Helfrich range, associated with the molecular tilt in the TC-SA, may be evaluated from their effects in the undulation of the membrane, accessible to the analysis of the U, P, and CU modes that do not monitor the orientation of the lipids.

Here, we introduce a Bedeaux–Weeks density correlation function^30^ method (BW-DCF) to obtain the bending and tilt moduli from MD simulations. Our approach does not require the construction of a mesoscopic surface or a tail-orientation field. Instead, it requires the number density profile ρ(*z*) of the phosphorus atoms in the lipid heads, and their density correlation function (DCF) *G*(*z*_1_, *z*_2_, *q*), Fourier transformed in the (*x*_2_ – *x*_1_, *y*_2_ – *y*_1_) coordinates. As in the DFT method,^26^ the Fourier transform for the DCF is obtained directly from the atomic positions of the lipids along the MD trajectories. Hence, we reduce the computational cost associated with the construction of surfaces and fields, and we circumvent the methodological issues related to the mapping of the smooth surface to perform Fourier transforms on a regular grid. Unlike the DFT method, our approach does not require the subtraction of lateral molecular correlations. This is achieved by considering the interlayer component of the DCF, that is, the correlation between the lipid densities in one and the other monolayer. The method proposed here relies on the rigorous theoretical analysis of Bedeaux and Weeks^30^ (BW), linking ρ(*z*) and *G*(*z*_1_, *z*_2_, *q*) to the mean square Fourier amplitudes of fluctuating interfaces. We introduce here a new element that links the interlayer component of the DCF to the coupled-undulatory (CU) mode.^19^ In addition, a deconstruction method, recently proposed and successfully tested for graphene sheets,^31^ is used to calculate the bending modulus κ, and other parameters describing the internal fluctuation modes of the membrane, such as the tilt-modulus κ_θ_ used in the tilt-based approaches.

We apply the BW-DCF method to coarse-grained and all-atom models of phosphatidylcholines (PC) lipids,^32^ which are widely used for in vitro experiments of synthetic membranes. We also evaluate the impact of cholesterol on the bending modulus of DPPC membranes. In section 2, we provide a short review of the methodological background (see original refs (19 and 30) and the SI for additional details). Sections 3.1 and 3.2 present the foundations and tests supporting the method. *The reader interested only in the practical use of the method may skip these sections and go directly to*section 3.3. Section 4 presents our results, compared with data obtained using previous methods. We finish the paper with a critical discussion and conclusions.

## 2 Computational and Theoretical Background

### 2.1 Molecular Dynamics (MD) Simulations

We tested the BW-DCF approach using molecular dynamics trajectories generated in our previous works.^19,33^ We simulated 1-palmitoyl-2-oleoyl-*sn*-glycero-3-phosphocholine (POCP) and 1,2-dipalmitoyl-*sn*-glycero-3-phosphocholine bilayers (DPPC) bilayers in water, modeled using the MARTINI force field.^34^ The intrinsic sampling method (ISM) used in our earlier studies^19^ relies on regular-grid Fourier transforms of the smooth surfaces corresponding to each lipid monolayer. Here, we perform a cross comparison of the results obtained with the ISM and the BW-DCF methods using exactly the same MD trajectories. This analysis provides a good reference to assess the accuracy of both methods.

We have performed in this work two additional simulations: (a) MARTINI molecular dynamics simulations of DPPC:Cholesterol with a (50:50) composition, corresponding to the *L*_o_ phase,^35^ and (b) full atomistic simulations of DPPC bilayers using the CHARMM36 force field.^36^ The simulations with the MARTINI model were performed at 320 K and those with CHARMM36 at 323.15 K for consistency with previous studies using the same atomistic force field.^37^ Furthermore, we performed simulations at zero and nonzero surface tensions. All the simulation details are discussed in the SI. A summary of the system sizes and applied surface tensions for each system are compiled in Table 1 of SI.

### 2.2 ISM and the Density Correlation Function

Both the ISM and BW-DCF methods use the coordinates of the phosphorus atoms in the lipid head groups, *r⃗*_*i*_ = (*x⃗*_*i*_, *z*_*i*_). We do not track any order parameters associated with the lipid aliphatic chains, such as their orientation or conformation. The ISM quantifies mesoscopic fluctuations of the two lipid monolayers by Fourier transforming the smooth surfaces^19^
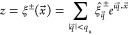
1which define the instantaneous mesoscopic shapes of the two (±) lipid layers, in terms of their Fourier components ξ̂_*q⃗*_^+^ and ξ̂_*q⃗*_^–^. Details of the ISM method are given in previous works^19^ and the SI of this paper.

The BW-DCF method does not require the intrinsic sampling, and it is therefore more computationally efficient. Instead, the BW-DCF approach requires the calculation of the phosphorus density profiles ρ(*z*) in the direction normal to the bilayer plane, and the Fourier transform of the density correlation function (DCF), *G*(*z*_1_, *z*_2_, *q*), on the bilayer plane, *XY*, where  defines the wavevector. The density profiles and DCFs are obtained as the usual statistical averages along the MD trajectory,
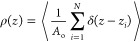
2where *A*_o_ is the projected area of the bilayer (*XY* plane) and
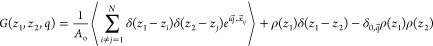
3where *x⃗*_*ij*_ = *x⃗*_*j*_ – *x⃗*_*i*_. The last term in eq 3, with Kronecker delta δ_0,*q*_, contributes only for *q* = 0. This term is not included in our analysis, since the BW-DCF method requires *q* > 0. The second term in eq 3 is independent of *q*, and represents the self-correlation term (*i* = *j*) excluded from the sum in the first term of eq 3.

The phosphorus density profiles (see Figure 1 in SI) consist of two symmetric peaks, which can be described by Gaussian functions, whose width increases with system size and decreases with the applied interfacial tension γ_o_. For membranes consisting of *N* ≲ 4000 lipid molecules the two (±) peaks are clearly separated, even in the tensionless state. Hence, the density profiles corresponding to each lipid layer can be resolved easily. We will denote the total density profile as ρ(*z*) = ρ^+^(*z*) + ρ^–^(*z*). For very large systems (*N* ≈ 8000 lipids) in the tensionless state, the density profiles of the two monolayers start overlapping with each other (see Figures 5 and 6 in SI). Even in this case, it is easy to evaluate the density contributions from each lipid layer, as the lipids do not undergo flip-flop motion during the typical times employed in our simulations, 0.1–1 μs. For the DCF, we considered *intralayer*, *G*^++^(*z*_1_, *z*_2_, *q*) = *G*^––^(*z*_1_, *z*_2_, *q*), and *interlayer*, *G*^+–^(*z*_1_, *z*_2_, *q*) = *G*^–+^(*z*_2_, *z*_1_, *q*), contributions.

The structure factor is a function of the 3D wavevector (|*q⃗*|, *q*_*z*_), defined by the Fourier transform of the DCF *G*(*z*_1_, *z*_2_, *q*) with respect to *z*_12_ = *z*_2_ – *z*_1_ and normalized by the number of lipid molecules
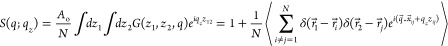
4We note that the BW-DCF approach uses *G*(*z*_1_, *z*_2_, *q*), and there is no need to evaluate the structure factor. Nevertheless, we use it here to test the validity of the main hypothesis of the method. Moreover, the structure factor provides a crucial link with X-ray and neutron diffraction experiments,^2,9,38,39^ when one accounts for the fact that the experimental data include the form factor with the scattering section of the full molecules, and contributions from the water bath. Unlike the experiments, BM-DCF includes only the phosphorus atoms located in the lipid polar heads and therefore the interlayer and intralayer contributions to the structure factor can be easily separated by calculating the sum in eq 3 over lipids in the same (intra) or different (inter) layers.

In the results presented below, we calculate the averages, eqs 2–4 using 5000 equilibrated and evenly spaced configurations along the MD trajectory.

### 2.3 Bedeaux–Weeks Theory

Bedeaux and Weeks^30^ (BW) analyzed the contribution of the interfacial fluctuations to the density correlation function *G*(*z*_1_, *z*_2_, *q*), expressing height fluctuations as density correlations. They used the Capillary Wave Theory (CWT) to describe the surface fluctuations, in terms of a *q*-dependent surface tension, γ(*q*). The main assumption in the CWT is that the Gaussian fluctuations acting on a surface *z* = ξ(*x⃗*), shift the local position of an intrinsic density profile ρ_I_(*z*). These surface fluctuations are characterized by the mean square values of the Fourier components ⟨|ξ̂_*q⃗*_|^2^⟩ and their dependence with the wavevector modulus *q* is often described through,
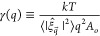
5interpreted as the free energy cost per unit of increased area, required to produce a corrugation with wavevector *q. γ*(*q*) can be obtained from the simulated ⟨|ξ̂_*q⃗*_|^2^⟩, using the ISM^19^ or alternative methods.

**Figure 1:**
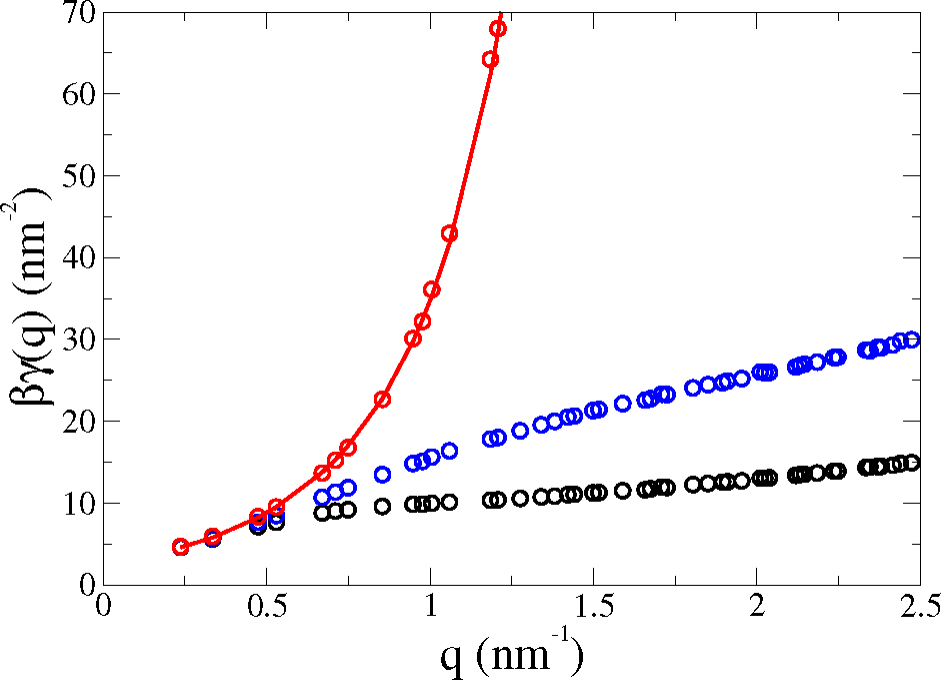
ISM-MD results for the surface tensions (in β^–1^ = *kT* units) as a function of the wavevector *q*, for a POPC membrane under tensile stress γ_o_ = 15.2 mN/m and *N* = 2000. The circles show the simulation results: (red) coupled undulatory mode γ^CU^(*q*); (blue) undulatory γ^U^(*q*), and (black) monolayer γ^m^(*q*). The red line is a guide to the eye.

In the range of *q*-vectors where the Helfrich surface Hamiltonian γ(*q*) = γ_o_ + *κq*^2^, applies.^40^ We note that using γ(*q*) as a generic function, rather than using just γ_o_ and κ, is important for the description of lipid membranes, since for *q* ≳ 0.3 nm^−1^, beyond the typical HH range, the complex elastic behavior of membranes requires additional elastic constants. As presented in Figure 1, different (*x*) definitions of the mesoscopic surface *z* = ξ^*x*^(*x⃗*) used in (5) result in quite different functions γ^*x*^(*q*).

Bedeaux and Weeks used a generic function, γ(*q*), to describe through eq 5 the fluctuations of the surface, *z* = ξ(*x⃗*) and obtain the CW contribution to the density correlation given by the series:
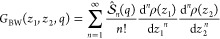
6where *n* denotes the order of the derivatives of ρ(*z*) and
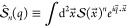
7is the Fourier transform of the *n*-power height–height correlation function . Since , all the coefficients  appearing in eq 6 can be calculated from γ(*q*).

The BW theory was originally developed for liquid surfaces, but it may be applied to any mesoscopic surface, ξ^*x*^(*x⃗*), and its corresponding γ^*x*^(*q*). We use here a truncated BW series by setting an upper limit *n* ≤ *n*_BW_ to the sum of eq 6. For *n*_BW_ = 1, we recover the Wertheim’s prediction of the density correlation function^41^
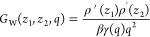
8where ρ′(*z*) is the derivative of the density profile. Eq 8 predicts a ∼*q*^–2^ divergence for *G*(*z*_1_, *z*_2_, *q*) at low *q*. This has been shown to be a reasonable approximation for the typical sizes employed in the MD simulations of liquid surfaces.^42^ However, for the lipid membranes studied here and also for graphene sheets,^31^ the contributions from higher-order terms are very important. Hence, to ensure convergence, we included up to *n*_BW_ = 20 terms in the calculations of the BW series. The *n* ≥ 2 terms are regular at *q* = 0 but they depend strongly on system size, while eq 8 is independent of the system size.

The BW series in eq 6 gives the contribution of the mesoscopic surface fluctuations to the DCF. The MD result for *G*(*z*_1_, *z*_2_, *q*), in eq 3 includes also contributions arising from fluctuations at molecular length-scales.^42^ We will refer to these contributions as the *correlation background*, *G*_b_ = *G* – *G*_BW_, which includes contributions arising from peristaltic fluctuations of the membrane thickness and the 2D compressibility modes of the lipid bilayer.

The BW expression for the structure factor *S*(*q*, *q*_*z*_) follows from the Fourier transform of *G*_BW_(*z*_1_, *z*_2_, *q*)
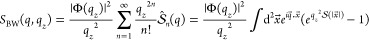
9where all the derivatives of the density profile are included in the *surface structure factor*
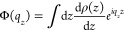
10The BW series can be generalized to include other fluctuation modes, like the coupled undulatory CU mode.^19^ In this case, *S*(*q*, *q*_*z*_) can be written in terms of intralayer and interlayer contributions, with Φ_+_(*q*_*z*_)Φ_+_(−*q*_*z*_)+Φ_–_(*q*_*z*_)Φ_–_(−*q*_*z*_) for intralayer and Φ_+_(*q*_*z*_)Φ_–_(−*q*_*z*_)+Φ_–_(*q*_*z*_)Φ_+_(−*q*_*z*_) for interlayer contributions. The intralayer term is a smooth function of *q*_*z*_, while the interlayer term is oscillatory, with a period 2π/*d*, where *d* is the distance between the head groups in the two lipid layers.

## 3 Bedeaux–Weeks Density Correlation Function (BW-DCF) Method for the Elastic Moduli of Lipid Bilayers

Sections 3.1 and 3.2 below, provide theoretical support and numerical validation of the main hypothesis used to develop the BW-DCF method. We discuss the ISM results for the different fluctuations modes of the lipid bilayer^19^ and compare the MD and BW results for the DCF and the structure factor. The reader interested in the practical use of the BW-DCF method may skip these sections and move directly to section 3.3, which contains eqs 13–16, describing the method to obtain γ^CU^(*q*) from the MD data (eq 3 for the DCF). The least-squares fit of these data to eq 19 gives access to the bending and tilt moduli, described in section 4.1.

### 3.1 Undulatory, Monolayer, and Coupled Undulatory Modes

In preparation for our discussion of the density correlation function, it is important to explore the dependence of γ(*q*) with the definition of the mesoscopic surface, *z* = ξ^*x*^(*x⃗*). Figure 1 shows the ISM results, eq 5, for γ^*x*^(*q*) using the undulatory mode (*x* = U) and the monolayer (*x* = m), as a function of *q*. Here we consider the *q*-values accessible in the simulation of a bilayer consisting of *N* = 2000 POPC lipids. The *q* = 0 term is not included in eq 5, but the data for γ^U^(*q*) and γ^m^(*q*) converge to the same *q* → 0 limit (see Figure 1).

The quadratic fit γ^*x*^(*q*) ≈ γ_o_ + *κq*^2^ to γ^U^(*q*) and γ^m^(*q*) obtained from the ISM-MD results might predict inaccurate bending moduli, κ, since γ^U^(*q*) and γ^m^(*q*) include contributions from protrusions. The coupled-undulatory mode (*x* = CU) was introduced^19^ to tackle this issue. The mean square average ⟨|ξ̂_*q⃗*_^*x*^|^2^⟩ in eq 5 was replaced by the interlayer correlation ⟨ξ̂_*q⃗*_^+^ξ̂_–*q⃗*_^–^⟩. The corresponding γ^CU^(*q*) does not feature the downward curvature observed in γ^m^(*q*) and γ^U^(*q*) (see Figure 1). The quadratic fitting to γ^CU^(*q*) provides a better estimate of κ, using the ISM-MD data at larger *q*. The difference between these functions at high *q* (wavelengths shorter than ∼20 nm) provide information on the peristaltic fluctuations of local membrane thickness.^19^ The CU description describes the decoupling of the two lipid monolayers with the rapid increase of γ^CU^(*q*), providing a natural upper limit for the wavevector range at which the membrane thermal fluctuations conform to those of a single surface.

### 3.2 Density Correlation Function and Its Mesoscopic Bedeaux–Weeks Representation

**Figure 2:**
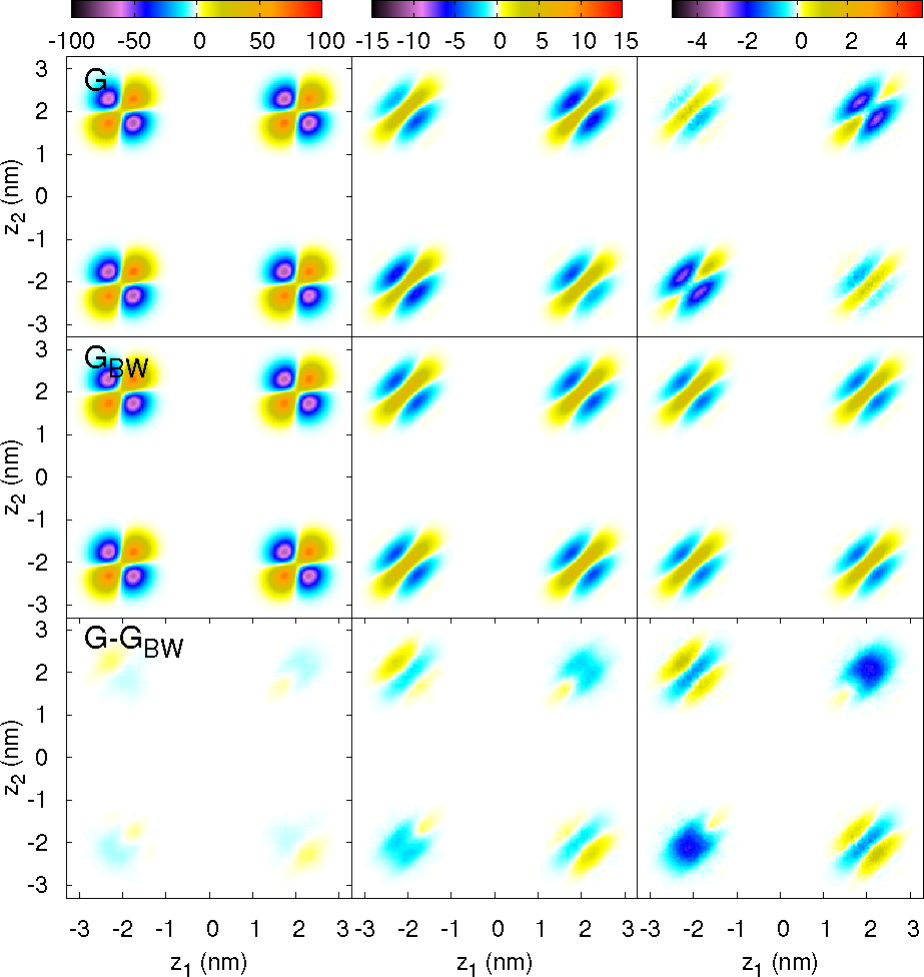
Maps for *G*(*z*_1_, *z*_2_, *q*), the Fourier transform on the *XY* plane of the density correlation function (DCF) in the POPC lipid membrane under tension γ_0_ = 15.2 mN/m and *N* = 2000, for three wavector values: Left column *q* = 0.237 nm^–1^, middle column *q* = 0.71 nm^–1^, and right column *q* = 1.18 nm^–1^. Top row: MD results. Middle row: Bedeaux–Weeks (BW) theoretical prediction using the undulatory mode (U) γ^U^(*q*), that is, the fluctuations of the mean surface between the two lipid monolayers. Bottom row: Difference *G* – *G*_BW_ that represents the correlation background, that is, molecular fluctuations missed by the mesoscopic BW description. Notice that the color scale (top) is kept fixed along each column, and the values of the correlation background are very similar for all the cases, but we keep the same color-scale along each column to better visualize the relative influence of that background with respect to the BW term.

In the following, we examine the general dependence of the density correlation function and assess the accuracy of the BW series to describe the simulation results both from the ISM results for γ^U^(*q*) and for γ^CU^(*q*). The top panels in Figure 2 represent the Fourier transform of the simulated DCF, *G*(*z*_1_, *z*_2_, *q*), for POPC bilayers under tension γ_0_ = 15.2 mN/m. We have represented results for three different wavevectors *q*. Note that the self-correlation term, ρ(*z*_1_)δ(*z*_1_ – *z*_2_) in eq 3 is not included in the plots. The left column in Figure 2 shows the results for the lowest nonzero wavevector (*q* = 0.237 nm^–1^) compatible with our MD simulation box size. This low *q* is in the pure undulatory regime, where γ^U^(*q*) ≈ γ^CU^(*q*) ≈ γ^m^(*q*) (see Figure 1). The right column in Figure 2 shows the results for a larger wavevector, *q* = 1.18 nm^–1^, corresponding to a weak coupling between the fluctuations of both lipid monolayers. In this regime, γ^*x*^(*q*) depends (see Figure 1) on the choice of *x* = U, CU or m. The middle column corresponds to *q* = 0.71 nm^–1^, an intermediate regime, with significant (but not full) coupling between the two lipid monolayers. We chose to present the DCF for a membrane under tension to isolate and better visualize the four quadrants describing the *intralayer* and *interlayer* contributions to *G*, that is, the correlations between lipids in the same or in different monolayers. For larger or tensionless membranes (see Figure 6 in SI), the four quadrants in *G*(*z*_1_, *z*_2_, *q*) would spread over a larger domain in the (*z*_1_, *z*_2_) regions and they could overlap with each other.

The theoretical mesoscopic predictions *G*_BW_(*z*_1_, *z*_2_, *q*) (middle row in Figure 2) were calculated using eq 6, with the derivatives of the mean density profile (at any order *n*) obtained from Gaussian fits to the density profiles of each individual lipid layer. We used the ISM function γ^U^(*q*) of the U mode to represent the fluctuations of the whole bilayer membrane. The numerical convergence of the BW series requires at least *n*_BW_ = 12 terms, and even up to *n*_BW_ = 20 terms, for larger systems or lower surface tension. The perfect symmetry of the four quadrants in *G*_BW_ emerges from the BW assumption that the intrinsic density profile of the whole bilayer follows strictly the undulations of the *z* = ξ^U^(*x⃗*) surface. Therefore, *G*_BW_ does not distinguish between intra and interlayer correlations. The left column of Figure 2 shows that this assumption is fairly accurate for the lowest *q*, for which the theoretical *G*_BW_ and the MD result for *G* are in very good agreement. In this regime, the main contribution to *G*_BW_ comes from the first (Wertheim’s) term in the BW series, eq 6. That term results in the vertical/horizontal boundaries between positive/negative values of *G* (within each quadrant), since the product ρ′(*z*_1_)ρ′(*z*_2_) changes sign at *z*_1,2_ = ±*d*/2. Increasing *q* (middle and right columns in Figure 2), the shape of the density–density correlation maps become skewed because the *n* ≥ 2 terms of BW series are more important and *G*_BW_(*z*_1_, *z*_2_, *q*) is not proportional to ρ′(*z*_1_)ρ′(*z*_2_). Within each quadrant, we find a sharp dependence on *z*_1_ – *z*_2_, with positive correlations for *z*_1_ – *z*_2_ ≈ 0, and negative correlations for larger |*z*_1_ – *z*_2_|. Along the other diagonal (i.e., changing (*z*_1_ + *z*_2_)/2), we observe a smoother dependence of *G*(*z*_1_, *z*_2_, *q*), without changes of sign.

We define the *correlation background* (b) as the difference *G*_b_ = *G* – *G*_BW_ between the MD results and the prediction of the BW series (see bottom row of Figure 2). This background includes all the density fluctuations that cannot be described as local shifts of an intrinsic density profile following the undulations of *z* = ξ^U^(*x⃗*), being the fluctuations due to the (2D-like) compressibility on each of two monolayers the main contributions to this background. The shape of *G*_b_ (as a function of *z*_2_ and *z*_2_) is very different in the intralayer and the interlayer quadrants. This indicates that the MD result for *G* does not have the perfect symmetry observed in *G*_BW_. The dependence of *G*_b_(*z*_1_, *z*_2_, *q*) with the transverse wavevector is very weak, the values for the three wavevectors *q* shown in Figure 2 are quite similar. Since the color scale in that figure is adjusted by columns, the color fading of *G*_b_ at the lowest *q* (left column) indicates that *G*_b_ becomes small in relation to the much larger *G*_BW_(*z*_1_, *z*_2_, *q*) contribution.

**Figure 3:**
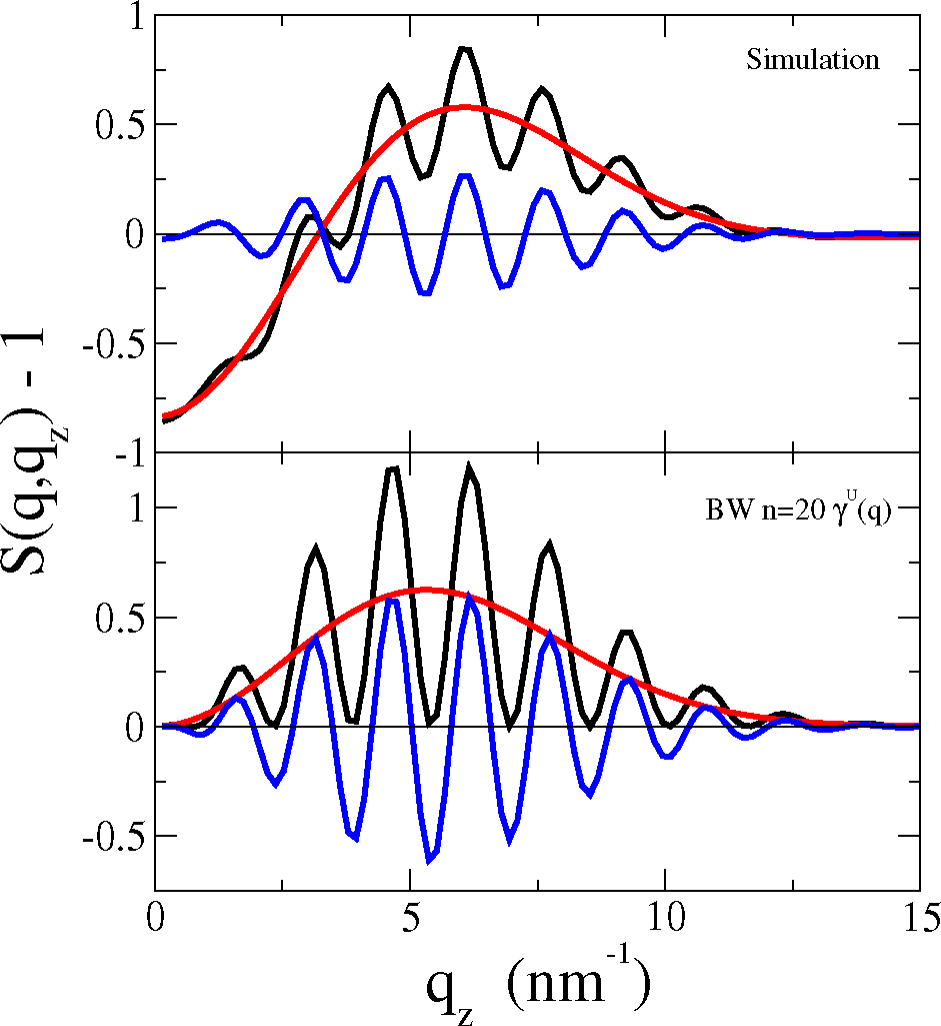
Interlayer structure factor *S*(*q*, *q*_*z*_) of the POPC under tensile stress γ_o_ = 15.2 mN/m and *N* = 2000 at *q* = 1.18 nm^–1^. Top panel: Direct MD results. Bottom panel: Bedeaux–Weeks (BW) theoretical prediction (see eq 9), using the undulatory mode (U) γ^U^(*q*). Black line: Total structure factor. Blue line: Interlayer component. Red line: Intralayer component.

To quantify the differences between *G* and *G*_BW_, we calculated the structure factors eq 4 and the corresponding BW prediction eq 9. Figure 3 presents *S*(*q*, *q*_*z*_) – 1 (black lines) and the contributions from the intralayer (*S*^+2^ + *S*^––^, red lines) and interlayer (*S*^+–^ + *S*^–+^, blue lines) quadrants in *G*(*z*_1_, *z*_2_, *q*), as functions of *q*_*z*_ at fixed *q* = 1.18 nm^–1^. The MD results (top) and the BW series (bottom) feature very different behavior. At *q*_*z*_ = 0 the BW series vanishes, as expected since Φ(0) = 0 for bilayer membranes, leaving only the trivial self-correlation value *S*_BW_(*q*, 0) = 1. In contrast, the MD result for *G* shows a negative *S*(*q*, *q*_*z*_) – 1 < 0, which gives the (background) density correlations within each lipid layer. The correlations from lipids in different layers show the same qualitative behavior in the MD and BW results, with oscillations ∼cos(*q*_*z*_*d*) reflecting the mean distance between the two lipid layers, and the broad envelop that vanishes both at low and high *q*_*z*_; but the amplitude of the oscillations is larger in *S*_BW_ than in the MD result for *S*. The symmetry of the quadrants in *G*_BW_ indicates that the envelop of the oscillations in *S*_BW_^+–^ + *S*_BW_^–+^ is equal to the smooth shape of *S*_BW_^++^ + *S*_BW_^––^ – 1. This does not apply to the MD results. At the transverse wavevector *q* = 1.18 nm^–1^ (the highest in Figure 2), *S*_BW_(*q*, *q*_*z*_) is quite different from the MD result *S*(*q*, *q*_*z*_), both at low and at intermediate *q*_*z*_ values. Lower values of *q* (as in the left and central columns in Figure 2) give much larger *S*_BW_, while the background contribution remains similar for all *q*, and the BW prediction becomes much more accurate to represent the full *S*(*q*, *q*_*z*_) (see SI).

To calculate the bending modulus κ from the DCF we have to interpret *G*(*z*_1_, *z*_2_, *q*) or *S*(*q*, *q*_*z*_) beyond their divergent contributions at low *q*, which are controlled just by the limit γ(0) = γ_*o*_;^31^ but at the same time, the contributions from the correlation background have to be small, that is, *G* ≈ *G*_BW_, because only in that case we may expect that the function γ(*q*) = γ_o_ + *κq*^2^ + ... (contained in the mesoscopic BW prediction) may be extracted from the full DCF given by the MD simulation. The results in Figure 3 show that we cannot fulfill these conditions by using the U mode as a description of the bilayer mesoscopic undulations. The background *G*_b_ is already visible for the smallest *q* and its relative weight in *G* grows fast with *q*. To extract κ from the simulated DCFs, we need to improve the mesoscopic description, that is, reduce the correlation background to ensure *G*_BW_ ≈ *G* over a wider *q* range. This problem is similar to what had been observed in the tilt-based DFT method.^26^

**Figure 4:**
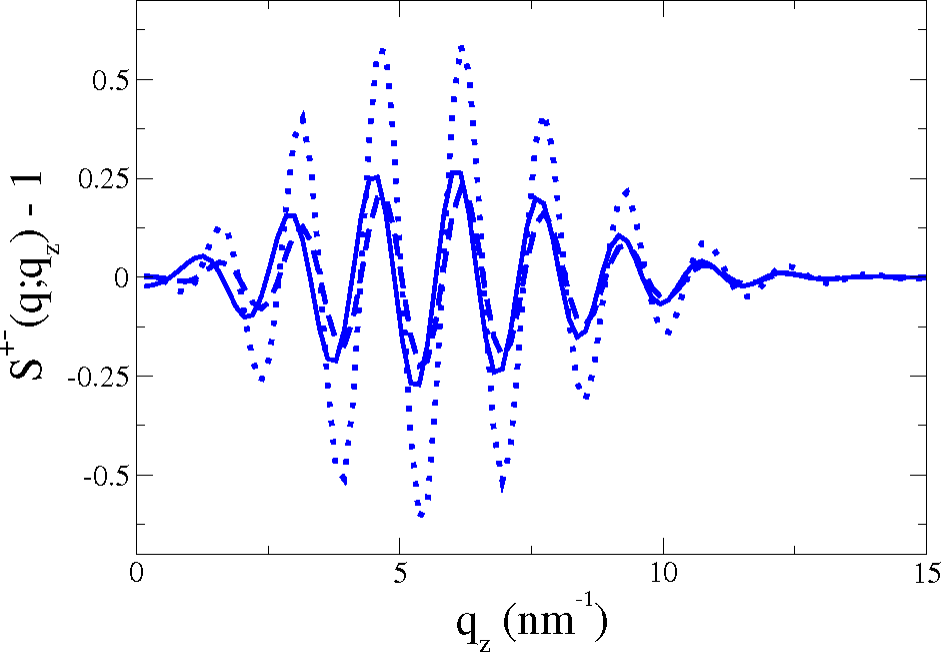
Interlayer structure factor *S*^+–^(*q*, *q*_*z*_) with *q* = 1.18nm^–1^ of the POPC under tensile stress γ_o_ = 15.2 mN/m and *N* = 2000. The MD result (full line) is compared with BW predictions *S*_BW_^+–^(*q*, *q*_*z*_) (calculated up to *n*_BW_ = 20 order) with γ^CU^(*q*) (dashed line) and γ^U^(*q*) (dotted line). In the SI, we show the same figure for the DPPC membranes.

The key to achieve a good agreement between the MD result eq 3 for the DCF and its mesoscopic description eq 6 is to focus on the interlayer component using the BW series and the CU mode. Using the ISM γ^CU^(*q*) and the density profiles of the two lipid layers, we get
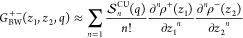
11and, from the Fourier transform, the corresponding interlayer structure factor *S*_BW_^+–^(*q*, *q*_*z*_). Figure 4 compares the interlayer structure factors obtained from this CU-BW approach (dashed line), the U-BW (dotted line), and the MD result (full line). Clearly, the CU-BW predictions are much closer to the MD results, and for wavevectors *q* ≳ 1 nm^–1^. Similar agreement between the CU-BW and MD results is obtained in tensionless POPC and DPPC bilayers (see SI).

In summary, Figure 4 shows that interlayer density correlations, with the CU version of the BW series, eq 11, provides the most accurate approach to model the simulated DFCs and the surface fluctuations described by γ^CU^(*q*). In our previous work,^19^ we concluded that the CU mode was the best approach to represent the HH regime for the fluctuations of the lipid bilayer membrane as a single surface. Here, we show that the *x* = CU mode γ^*x*^(*q*) is the best choice to link the surface fluctuations with the DCF.

### 3.3 BW-Deconstruction of the MD Interlayer DCF to Get γ^CU^(*q*)

To calculate the DCF using the BW series (eqs 6,11)) one needs the density profiles ρ(*z*) = ρ^+^(*z*) + ρ^–^(*z*) and the mean square surface fluctuations, γ^x^(*q*). Assuming that this mesoscopic prediction describes accurately the simulation result, it should be possible to obtain a function γ(*q*) that fulfills *G*_BW_ ≈ *G*. This would circumvent the need to compute the surfaces *z* = ξ^±^(*x⃗*) with the ISM, for each molecular configuration. We have shown that for lipid membranes, this task should be possible by considering the interlayer component of the DCF, that is, the correlations between lipid molecules in opposite sides of the bilayer membrane, which gives γ^CU^(*q*).

A difficulty in the implementation of the approach proposed here is that one needs to define a method to *deconstruct* the full BW series eqs 6 and 11) and obtain γ^CU^(*q*) from the DCF. For liquid surfaces, this problem has often be addressed using the *n* = 1 (Wertheim’s) term in the BW series. This approach gives a DCF contribution eq 8 that can be easily inverted using the first derivative of the density profile. However, to describe the DCF in a lipid bilayer one must include many other terms in the BW series to ensure convergence. In that case the function γ^CU^(*q*) will appear in a convoluted way in *G*_BW_^+–^(*z*_1_, *z*_2_, *q*), through the functions *S*_*n*_^CU^(*q*). A *deconstruction* method to extract the full function γ(*q*) from the simulation result for *G*(*z*_1_, *z*_2_, *q*) has been successfully implemented for graphene sheets.^31^ We adapt this method here to study lipid membranes.

We assume that the MD interlayer DCF *G*^+–^(*z*_1_, *z*_2_, *q*) has the functional form given by eq 11 and project it on the *n*–order derivatives of ρ^+^(*z*_1_) and ρ^–^(*z*_2_) for any *n* up to a value *n*_BW_. These derivatives are calculated using a Gaussian fit , with the 2D density ρ_o_, the mean thickness of the membrane *d*, and the mean square width α obtained from the simulated ρ(*z*) (see eq 2). Notice that any global displacement of the bilayer is eliminated by setting the center of mass of all the phosphorus atoms as the origin *z* = 0 for each molecular configuration.

With the Gaussian fit, the derivatives at any order may be evaluated with the recursion relation:
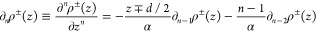
12Then, we define two *n*_BW_ × *n*_BW_ matrices,  and , with elements
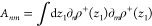
13and
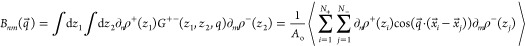
14that uses the atomic coordinates along the MD simulation, with the index *i* running over the lipid molecules of one layer and the index *j* over the other. The first line in eq 14 requires the previous calculation of *G*^+–^(*z*_1_, *z*_2_, *q*) from the MD trajectory, with a binning for *z*_1_ and *z*_2_. Alternatively, we may use the second line in eq 14 to calculate directly the contribution of each lipid pair to *B*_*nm*_(*q⃗*), without storing *G*^+–^(*z*_1_, *z*_2_, *q*). The accuracy of the statistical sampling along the MD simulation may be assessed from the results for the different wavectors with the same modulus *q* = |*q⃗*|, which are accumulated in a mean .

From eq 11 we get the matrix equation , where  is a diagonal matrix with elements
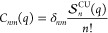
15that may be obtained through the inverse matrix , as . Note that  is independent of *q*, and its inverse has to be calculated only once.

Since , the wavevector dependent surface tension of the CU mode follows directly from the *C*_11_(*q*) element of the matrix , as
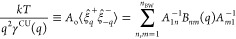
16The accuracy of the method depends on the truncation of the series at a finite order, *n* ≤ *n*_BW_, the BW series in eq 11. To achieve convergence the results for γ^CU^(*q*), the minimum number of terms in the series should increase with the system size, while it may be decreased for increasing γ_o_. The requirement for using many terms in the series, up to *n*_BW_ ≈ 12, to achieve robust results for γ^CU^(*q*) highlights the importance of using the full Bedeaux and Weeks theory (eqs 6 and 11), rather that the much simpler Wertheim’s relation eq 8 that describes the DCF just in terms of the first derivative of the density profile.

## 4 Results

**Figure 5:**
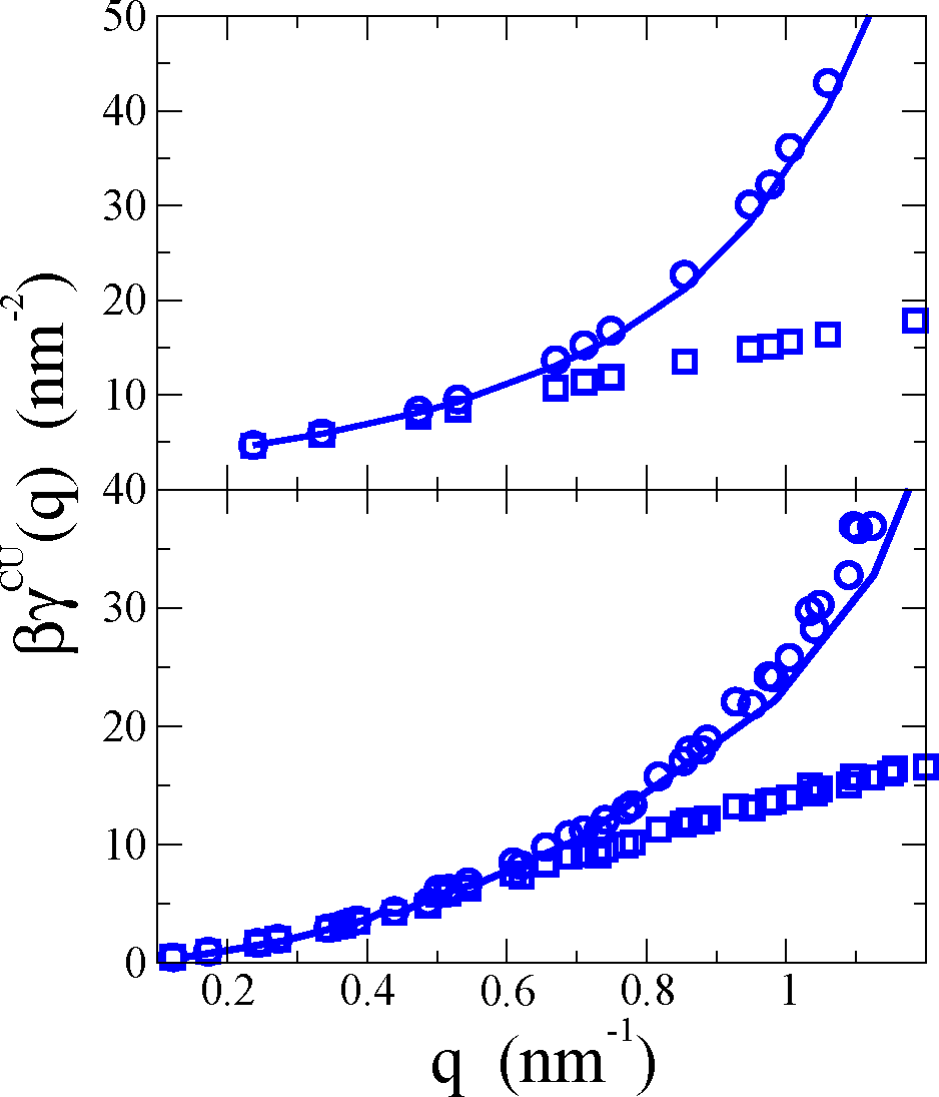
Wavevector dependent surface tension γ^CU^(*q*). Top panel: POPC under tension γ_o_ = 15.2 mN/m with *N* = 2000 and bottom panel, tensionless POPC with *N* = 6000. The full lines represent the results from the BW-*deconstruction* (using *n*_BW_ = 20 terms of the series) of the MD results for *G*_+–_(*z*_1_, *z*_2_, *q*). The symbols represent the ISM-MD results: circles, γ^CU^(*q*), and squares, γ^U^(*q*).

**Figure 6:**
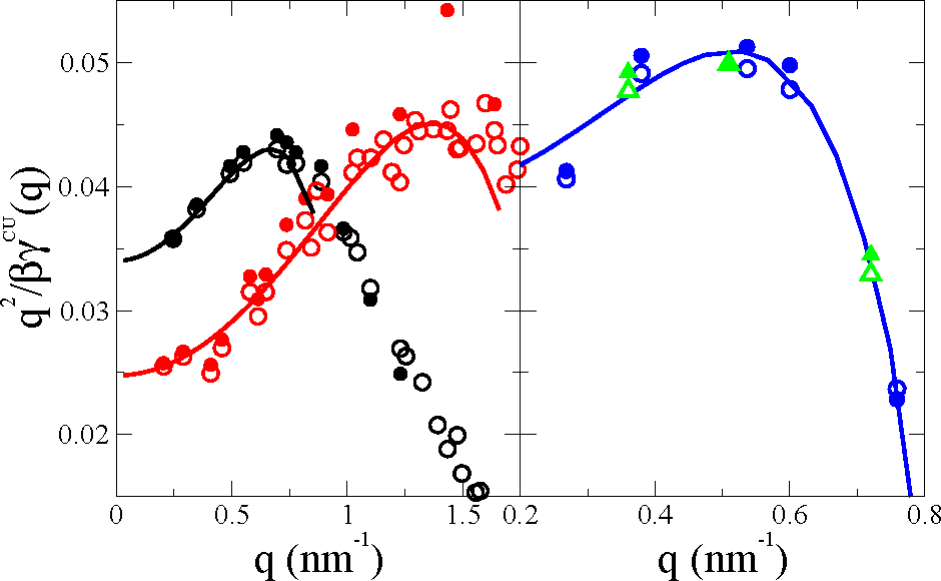
Inverse of the coupled undulatory surface tension, γ^CU^(*q*), for the DPPC membranes analyzed in this work. Left panel: MARTINI tensionless DPPC with *N* = 2048 (black), and MARTINI tensionless DPPC–cholesterol with *N* = 2304 (red). Right panel: CHARMM36 all-atom simulations of tensionless DPPC with *N* = 1800 (blue) and *N* = 1000 (green) The symbols represent the simulation data for the ISM results (empty symbols) and the results from the BW deconstruction BW to order *n*_BW_ = 20 of the simulated data, *G*_+–_(*z*_1_, *z*_2_, *q*) (full symbols). The solid lines show the fittings to the ISM (for the all-atoms *N* = 1800 of the BW) data to eq 19 in the range 0 < *q* < 0.9*q*_*d*_. The same figure for the POPC membranes is shown in SI (see Figure 13). Numerical data for the fitting coefficients are collected in Table 1.

The results for γ^CU^(*q*) are presented in Figure 5 for the same POPC lipid membrane as in Figures 2–4 (top panel), and for tensionless POPC membranes of different sizes (bottom panel). For tensionless membranes the low *q* behavior is better analyzed^21^ through *q*^2^/γ^CU^(*q*) (see Figure 6), which is used to estimate κ^–1^ from the extrapolation to *q* = 0. In all cases we compare the results from the ISM identification of the mesoscopic surfaces *z* = ξ^±^(*x⃗*) and those from the deconstruction of BW series eq 16 for the simulated DCF eq 3. For all systems and all sizes, we observe very good agreement between the ISM and the BW-DCF results, up to wavevectors *q* ≲ 1 nm^–1^. This transverse wavevector is well below the value *q*_*u*_ ≈ 2.6 nm^–1^ for which the CWT assumptions for the intrinsic density profile were valid, but here, we are asking the theory for a stronger requirement on the DCF, and we cannot expect that the condition *G*_BW_ ≈ *G*, still holds when γ^CU^(*q*) becomes very large and therefore *G*_BW_ decays below the background. The results for γ^CU^(*q*) with other systems and force fields are shown in the SI (see section VI).

### 4.1 Bending and Tilt Moduli Obtained from the BW-DCF γ^CU^(*q*)

We have shown above that γ^CU^(*q*) can be calculated over the discrete set of wavevectors (2π/*L* ≤ *q* ≲ 1 nm^–1^) either using the ISM^19^ or the BW-DCF method proposed here eq 16. Since in the MD simulations with the MARTINI force field we have used rather large systems (up to 6000 lipid molecules), we could in principle calculate the bending modulus κ by using a least-squares fitting γ^CU^(*q*) ≈ γ_o_ + *κq*^2^. However, such fitting will in general underestimate the true bending modulus, because for tensionless membranes, and the system sizes employed in the simulations, the fitting gives too little weight to the wavevector domain 2π/*L* ≤ *q* ≲ 0.3 nm^–1^, that is truly representative of the Helfrich Hamiltonian regime.

The tilt-curvature approaches^20−23,43^ have highlighted the effects beyond the *κq*^4^ energetic cost of the softest fluctuation mode tensionless membranes at low *q*. In that mode the molecules follow the undulations of the surface *z* = ξ(*x⃗*), with the lipid hydrocarbon tails adopting a local orientation normal to the surface. Hence, the chains are locally tilted with respect to the direction **ẑ** normal to the mean plane of the membrane. A different fluctuation mode that keeps the molecules along **ẑ** (without local tilt) has an energetic cost κ_θ_*q*^2^, where κ_θ_ is a new parameter with units of surface tension.^44^ At , that is, in the Helfrich’s regime, the true bending modulus κ describes the softest fluctuation mode. At , the untilted mode may be softer and therefore more relevant, and γ(*q*) ≈ κ_θ_*q*^2^.

The usual approach to obtain an accurate result for κ in tensionless membranes is by considering the low *q* limit of . The tilt modulus κ_θ_ appears as the quadratic term in the expansion of the undulatory mode^21,22,44^
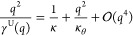
17If we use the ISM results for the U mode, this (truncated) expansion may be used to get a good fit over a broad range of *q* beyond the HH regime, and to determine κ. Although we did not do it in our earlier ISM analysis,^19^ the coefficient κ_θ_ may also be identified over the wavevector range 0.3 nm^–1^ ≲ *q* ≲ 0.6 nm^–1^, and we may even get a quartic coefficient (as used in the most recent TC-SA analysis^23^). Notice that in the ISM approach we keep track only of the lipid head groups, not of the lipid tail orientations. Hence, we do not obtain κ_θ_ in eq 17 by fitting the simulated tail orientations. However, very different approaches, theoretical and experimental,^45^ provide evidence for the need of including a *q*^2^/κ_θ_ term in eq 17 and give similar values for the coefficient κ_θ_.

The BW-DCF route introduced in this work, eq 16, is not applicable to the undulatory mode, γ^U^(*q*), since it contains a large contribution from the non-BW background to the DCF. Hence, only the CU mode may be obtained directly from the interlayer DCF because it has very little correlation background over its BW series. The U and CU modes are identical up to the quadratic expansion (eq 17), since
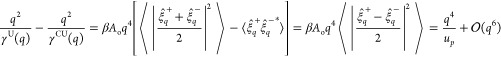
18in terms of the elastic spring constant *u*_P_ = [*βA*_o_⟨(*d* – ⟨*d*⟩)^2^⟩]^−1^ for the *q* → 0 limit of the peristaltic (P) fluctuations in the membrane thickness.^19^ However, as shown in Figure 6, the shape of *q*^2^/γ^CU^(*q*) is much more complex than the (nearly) quadratic result for the U mode. Their difference, from the *q*^4^ and higher order terms in the expansion, emerges from the gradual decoupling of the fluctuations of the two lipid layers as *q* increases. We may define a function, , to quantify the decoupling. Then, the CU version of eq 17 is
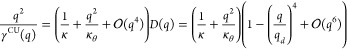
19where a single parameter *q*_d_ includes the *q*^4^ terms in *D*(*q*), to represent the typical *q* value at which the two monolayers in the membrane become uncoupled. The full shape of *D*(*q*) may be obtained from the ISM analysis of the U and CU modes, showing that *D*(*q*) goes to zero in the large *q* limit (see SI for details). However, the simple parametric form (eq 19) allows us to extract κ, κ_θ_, and *q*_*d*_ directly from the BW analysis of the interlayer DCF. The three parameters can be extracted from a least-squares fit in the range 2π/*L* ≤ *q* ≲ *q*_*d*_. Hence the minimum practical system size we can use with the BW-DCF analysis is determined by the value of *q*_*d*_. As commented below and in SI, the parametrization (eq 19), with the explicit use of the tilt mode elastic constant κ_θ_, becomes more accurate and generic than our earlier proposal^19^ developed and tested with the ISM for pure lipid membranes and the MARTINI force field.

### 4.2 Comparison with Previous Results

**Figure 7:**
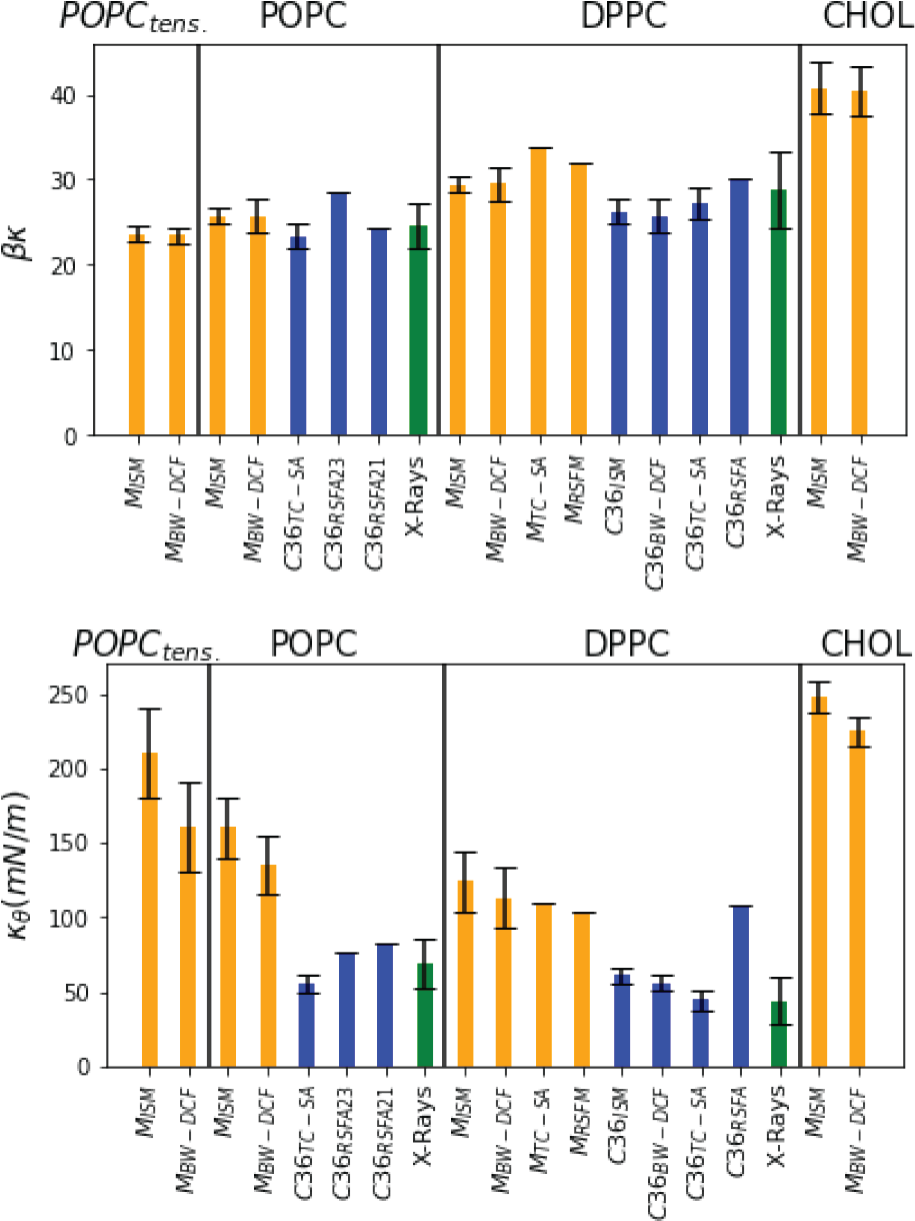
Visual representation of the values of bending κ and tilt κ_θ_ modulus shown in Table 1. Orange, blue, and green highlight results obtained with MARTINI, all-atom force field, and X-ray experiments.

We illustrate our BW-DCF method, eqs 16–19, by analyzing MD trajectories of DPPC membranes described with two different force fields: coarse grained MARTINI and all-atom CHARMM36. We have also used the MARTINI force field to simulate tensionless POPC membranes, POPC membranes under tension, and a DPPC–cholesterol mixture with 1:1 composition in DPPC:CHOL. For all the cases presented in Figures 5 and 6 and Table 1 and in SI, we compare the BW-DCF analysis for γ^CU^(*q*), from eq 16 with the results obtained with the ISM.^19^ The ISM gives direct access to both γ^U^(*q*) and γ^CU^(*q*), over a longer *q* range, but it requires the definition and calculation of the smooth surfaces representing the instantaneous shape of the lipid membrane. In contrast, the γ^CU^(*q*) can be obtained directly from the simulated density profiles and density correlations with the BW-DCF method, using a straightforward approach, summarized by eqs 14–16 and a least-squares fit to eq 19. The error-bars in Table 1 and Figure 7 are estimated considering: (a) the quality of the statistical sampling, which may be assessed by the difference between the results of γ^CU^(*q*) for different *q⃗* with the same modulus *q* and (b) the set of *q* values chosen for the least-squares fit, which depend on the system size and the value of *q*_d_. Both (a) and (b) can be systematically improved (as with any other analysis method) at the computational cost of using larger system sizes and longer simulation times. However, we note that the BW-DCF approach is free of methodological uncertainties associated with the use of mesoscopic height and tilt fields.

**Table 1:** Bending Modulus κ and Tilt Modulus κ_θ_ for POPC and DPPC Bilayers Simulated with the Coarse-Grained MARTINI and the All-Atom CHARMM36 Force Fields^a^

*N*	model	method	*βκ*	κ_θ_ (mN/m)	*q*_d_ (nm^–1^)
POPC under tension *βγ*_0_ = 3.39 nm^–2^					
2000	MARTINI	*M*_ISM_	23.6 ± 1	210 ± 30	1.07 ± 0.05
		*M*_BW-DCF_	23.4 ± 1	160 ± 30	1.07 ± 0.05
POPC free					
6000	MARTINI	*M*_ISM_	25.7 ± 1	160 ± 20	1.17 ± 0.05
		*M*_BW-DCF_	25.7 ± 2	135 ± 20	1.14 ± 0.05
648	CHARMM36	*C*36_TC-SA_^23^	23.3 ± 1.4	50.6–62.1	
416	CHARMM36	*C*36_RSFA_^10^	28.4	76.8	
416	CHARMM36	*C*36_RSFA_^24^	24.3	82.0	
stacks		X-rays^9^	24.6 ± 2.6	69 ± 17	
DPPC free					
2048	MARTINI	*M*_ISM_	29.4 ± 1	124 ± 20	1.15 ± 0.05
		*M*_BW-DCF_	29.5 ± 2	113 ± 20	1.07 ± 0.05
1800	CHARMM36	*C*36_ISM_	26.2 ± 1.5	61 ± 5	0.82 ± 0.05
		*C*36_BW-DCF_	25.7 ± 2	56 ± 5	0.82 ± 0.05
648	CHARMM36	*C*36_TC-SA_^23^	27.2 ± 1.8	37.0–51.4	
2048	MARTINI	*M*_TC-SA_(22)	33.7	110	
416	CHARMM36	*C*36_RSFA_^24^	30.1	108.3	
2048	MARTINI	*M*_RSFM_(10)	31.9	103.2	
stacks		X-rays^9^	28.8 ± 4.5	44 ± 16	
DPPC cholesterol free					
2304	MARTINI	*M*_ISM_	40.8 ± 3	248 ± 10	2.0 ± 0.2
		*M*_BW-DCF_	40.4 ± 3	225 ± 10	2.2 ± 0.2

a *N* represents the total number of lipids, and *βγ*_0_ is the surface tension applied to the membrane. The calculations with the ISM and BW-DCF were performed fittings the coupled-undulatory γ^CU^(*q*) mode (eq 19).^19^*q*_d_ represents the decoupling parameter. We compare our results with experimental data obtained using X-rays,^9^ and previous simulations, using tilt-curvature methods (TC). The “Spectral Analysis” (TC-SA) results were obtained using the height spectra (κ) and the lipids director fluctuations (κ_θ_), with atomistic^23,46^ and coarse-grained^22^ force fields. We also list results obtained with the Real Space Instantaneous Surface Method for atomistic (RSF_A_)^10,24^ and coarse-grained simulations (RSF_M_).^10^

The ISM and BW-DCF results for the bending modulus κ are always very close to each other (within one percent) and feature clear trends among the simulated systems (see final section for a discussion). The results for κ_θ_ also very similar, albeit they have larger uncertainties. This is due to the similar values of *q*_d_ and the tilt-threshold , which leads to a mixing of modes that introduced uncertainty in the fitted values of κ_θ_ and *q*_d_. The simulations of a DPPC–cholesterol membrane (at 1:1 concentration) provide a good example of a system where these two effects are well separated. The high concentration of cholesterol results in an increase of the bending and tilt moduli with respect to the pure DPPC membrane, but  remains approximately the same in the pure DPPC (0.93 nm^–1^) and DPPC:CHOL bilayers (1.12*n*^–1^). However, in the membrane with cholesterol the value of *q*_d_ is twice as large as in the pure DPPC membrane. At least within the MARTINI model, the cholesterol molecules influence the fluctuations of the two DPPC layers, which remain coupled over a range 1 nm^–1^ ≲ *q* ≲ 2 nm^–1^, which is dominated by the tilt modulus, instead of the bending modulus. Again, for pure DPPC or POPC membranes the thresholds for the ± decoupling and tilt-bending are very similar. The need to improve our earlier empirical proposal^19^ for a fit to the CU, which did not include an explicit κ_θ_ parameter, became evident with the effects of cholesterol in the DPPC MARTINI model.

The bending modulus predicted by the MARTINI and all-atom CHARMM36 force fields are similar. We also find reasonable agreement with previous computations by other authors (see Table 1 and Figure 7). Our κ_θ_ results for pure DPPC bilayers do, however, feature significant differences for CHARMM36 and MARTINI force fields, even when we account for the uncertainties in the results, which are significant for the MARTINI model. This difference might be associated with the very different representation of the aliphatic chains in coarse grained and atomistic models. Establishing this as the origin of the difference reported here requires additional work. We note that previous simulations using the RSF_A_ and RSF_M_ approaches reported smaller differences in the tilt modulus obtained with CHARMM36 and MARTINI force fields (c.f., DPPC free *N* = 416 and *N* = 2048 results in Table 1), although the system sizes employed were very different. Indeed, the system size required to obtain a good estimate of the elastic constant is expected to depend on the specific force field employed. For DPPC membranes modeled using all-atom CHARMM36 the value of *q*_d_ is ∼25% lower than in MARTINI, that is, the limited flexibility of the coarse-grained molecular tails maintains the two lipid monolayers correlated with each other over a longer *q* range. Consequently, to achieve a similar fitting accuracy using eq 19, the simulations with CHARMM36 need to be performed with larger systems than in the case of MARTINI. The *N* = 1800 system molecules reported in Table 1^1^ is close to the smallest size that may be practically used with all-atoms force field to obtain elastic constants with a 5% precision. Another practical issue we have found in our MD simulations, which should apply to any method, is concerned with the atomistic description of the lipids, which results in low-*q* fluctuation modes that are much slower than the corresponding one in the coarse-grained MARTINI force field. Hence, the all-atom force fields require the use of both larger systems and longer runs, as compared with coarse-grained force fields. Although, our all-atom simulations involve trajectories spanning 660 ns for the *N* = 1800 membrane, we expect that the statistical sampling of the lowest *q* modes might still be improved by performing longer runs.

We compare in Table 1 and Figure 7 our results for the POPC and DPPC membranes, with previous simulations by other authors. Those simulations were obtained different tilt-curvature (TC) approaches: (a) The real space functional (RSF) method,^24^ which has been used to study very small system sizes containing few hundred lipids. This method was developed as an alternative to Fourier transform methods, which require larger systems sizes. The RSF method has been applied to study POPC and DPPC membranes and the MARTINI force field,^10^ as well as membranes modeled with the all-atom CHARMM36 force field.^10,24^ (b) The spectral analysis (TC-SA), which uses a regular-grid Fourier transform for the mesoscopic surface (as the ISM) and for the tail-orientation field. The most advanced version of this method has been applied to POPC and DPPC bilayers using the all-atom CHARMM36 force field,^23,46^ while an early version of the method was applied to coarse-grained MARTINI membranes.^22^

The results obtained with the TC methods depend on the mesoscopic definition and on the specific choice for the analysis. The most recent TC analysis^23^ give different values for κ and κ_θ_, obtained from the height or from the tilt spectrum, and under one or another hypothesis. Our BW-DFC (and ISM) results show better agreement with the TC-SA data. The SA version of the TC method relies on the analysis of the height spectrum with a κ_θ_ obtained normalizing to unit the *z* component of the tilt vector. As discussed above the BW-DFC method proposed here is more computationally efficient, since it does not require the construction of a height and tilt smoothed fields. The bending moduli might change depending on the criterion employed to construct the fields. This uncertainty is not present in the BW-DCT, and this represent in our view a definite advantage of the method.

Regarding the results obtained with the RSF methods, the bending modulus appears to be overestimated by about 10%, with respect to the TC-SA and our BW-DCF and ISM results. The difficulty of RSF method to get κ_θ_ has been discussed before^24^ and this is reflected in the inability of this approach to reproduce the important differences (up 100% increase) between the MARTINI and the CHARMM36 force fields. See for instance our results for BW-DCF or ISM for DPPC “free” bilayers (and the results using TC-SA), with the MARTINI force field predicting a much higher κ_θ_. Instead, the RSF approaches predict essentially the same κ_θ_.

The X-ray results for κ and (with larger error bars) for κ_θ_, based on the analysis of the height–height fluctuations,^9^ are in fair agreement with our results for CHARMM36-DPPC and also with the results obtained with latest version of the TC-SA method. Clearly the all-atoms force field are accurate predicting the elastic properties of phospholipid membranes, considering uncertainties of the experimental and computational results. Also, note that the MARTINI force field provides a good estimate of the bending modulus but, based on our results, clearly overestimates the tilt modulus.

## 5 Discussion

The state of the art methods to compute the bending modulus of in silico lipid bilayers rely on the construction of mesoscopic height-surfaces^19^ and tilt-vector fields^20−23,43^ as key steps to predict membrane elasticity from molecular degrees of freedom. The application of these mesoscopic descriptions requires the removal of many molecular details, hence opening several questions on what degrees of freedom must be discarded, and what theoretical framework must be used to analyze mesoscopic fields using a small set of elastic constants.

A recent review on the tilt-curvature spectral analysis (TC-SA)^23^ provides an illuminating discussion of the key problems associated with the computation of bending moduli and how these problems motivate the development of theoretical methods, which can predict disparate bending moduli (see Table 2 in ref (23)). In order not to have to subtract the nontrivial lateral correlations, the TC-SA descriptions have to use smooth mathematical representations for the instantaneous membrane shape and Fourier transform them using a regular grid. To circumvent the use of regular grids, the so-called direct Fourier transform (DFT) method^26^ relies on the analysis of atomic positions in real space. Such analysis can be used to obtain the density correlation function (DCF) of the lipid molecules. A link between the bending modulus, κ, and the DCF was suggested a long time ago,^47^ but a practical implementation was missing. The DFT and any direct use of the DCF to get κ face the difficulty to separate the lateral correlation structure from the height–height correlations.^27^

We have proposed in this article an alternative method, eqs 16–19, that relies on the analysis of the DCF extracted directly from molecular dynamics (MD) simulations (eq 3). The main ingredients of our method are (a) the Bedeaux–Weeks (BW) theory for the DCF of fluctuating interfaces eq 6 and (b) the coupled undulatory (CU) mode introduced in ref (19). The CU mode disentangles the mixing of mesoscopic undulations from fluctuations appearing at high-*q* modes, such as lipid protrusions. Within the CU approach, we have set a limit, *q* < *q*_d_ in eq 19, for the wavevector range corresponding to correlated fluctuations of the two lipid monolayers. The BW theory, designed initially to describe liquid surfaces, might be adapted to link the CU fluctuations in a lipid membrane with the interlayer component of the corresponding DFC. In this way, we circumvent the difficulties associated with the initial DFT approach^23^ since the DCF of lipid molecules in different layers provides a natural filter for the lateral correlation background. The BW-DCF method presented here eliminates the computational cost and, more importantly, the conceptual difficulties associated with the definition of mesoscopic height and tilt fields. In addition, the BW-DCF method only requires selecting an atom in the lipid head as the descriptor of the lipid position. Here, we showed that the phosphorus atom provides a good choice in POPC and DPPC lipids. We expect that other choices of atoms in the lipid head groups would have little impact on the results presented here (see Figure 6 in the Supporting Information of ref (19)).

We have discussed the foundations of the BW-DCF method, proposed here to computer bending moduli. The good agreement between the description of the CU modes by ISM and BW-DCF, as reported here, supports the accuracy of our hypotheses. The major advantages of the BW-DCF method are the ease of implementation, higher computational efficiency, and reduction of the uncertainty in the computed results, associated the use of specific criteria to define the undulating surfaces. The function γ^CU^(*q*) can be directly obtained from eqs 14–16. The bending, κ, and tilt moduli, κ_θ_, follow from a least-squares fit to eq 19.

A potential limitation of the BW-DCF method is that, since it relies on the CU mode, it cannot be used to describe the elastic properties of the membrane at *q* higher than the decoupling threshold given by *q*_*d*_ in eq 19. The U mode, that is, the fluctuation of the mean surface between the two lipid monolayers, may be used for higher wavevectors. However, we may question its physical interpretation as “membrane undulations” for very high-*q* values since, in that regime, the fluctuations of the lipid monolayers are almost entirely uncorrelated. In contrast, our function γ^CU^(*q*) consider that the description of the membrane as a single sheet, should be limited to the domain *q* ≲ *q*_*d*_.

## 6 Conclusions

We have introduced the BW-DCF methodology to compute the bending modulus of lipid bilayers, eqs 16–19. We have illustrated the method by analyzing molecular dynamics trajectories of POPC and DPPC lipid membranes using coarse-grained and fully atomistic force fields. We have investigated membranes under tension and in a tensionless state. In addition to pure bilayers, we investigated mixed DPPC: cholesterol bilayers at 1:1 composition. We conclude the following:

- The BW-DCF predicts bending moduli in excellent agreement with results obtained using the more involved intrinsic sampling method (ISM). In contrast to BW-DCF, the ISM method requires the construction of intrinsic surfaces for each configuration and performing the corresponding fixed-grid Fourier transform. The BW-DCF results agree, within the uncertainty of our computations, with earlier simulations using the tilt-curvature spectral analysis (TC-SA),^23^ which also requires the definition of a tilt vector field.
- The results obtained with the coarse-grained MARTINI force field should be taken with caution since the comparison with all-atoms CHARMM36 show that the MARTINI force field overestimates κ by about 8% and κ_θ_ by a much larger amount (it can be up to 100%). While the bending modulus could be improved by adjusting the force field parameters, the differences in κ_θ_ might reflect the significantly different flexibility of lipid molecules modeled with coarse-grained or all-atom force fields. This is an important aspect that requires further investigation. We note that the simplest (real-space) methods using the tilt-curvature analysis^10,24^ do not reproduce the differences observed between coarse-grained and fully atomistic models. Hence, these methods require revision.
- Our CHARMM36-DPPC results are in fair agreement with those obtained with the most advanced TC-SA versions.^23^ The experimental X-ray data for DPPC stacks^9^ are in very good agreement with our CHARMM36 results for κ and well within the error bars for κ_θ_.
- The coarse-grained force field overestimates also the *q*-vector range *q* < *q*_*d*_ for correlated fluctuations of the two lipid layers. Accurate estimates of κ and κ_θ_ using the all-atom CHARMM36 force field can only be obtained using lower *q* values. In practice, accessing these low *q* vectors requires system sizes for all-atom force fields (*N* ≥ 1500) that are larger than those employed using the MARTINI force field.

On the basis of our simulations of the MARTINI force field, we also conclude the following:

- POPC membranes are more flexible than DPPC ones. POPC gives κ ∼15% lower and κ_θ_ ∼20% higher than DPPC. Upon application of tension, the POPC bending decreases, ∼8% for surface tension of ∼15 mN/m. However, κ_θ_ may be larger in the tensed membrane.
- Addition of cholesterol to DPPC bilayer in a concentration (1:1), corresponding to *L*_o_ phase regime results in a significant increase in the bending and tilt moduli. Moreover, cholesterol acts as a linker between the lipid layers. We find that the range of correlated fluctuation expands to much higher vectors *q* ∼ 2 nm^–1^ than in pure bilayers (∼1 nm^–1^).

The points listed above are important for developing better force fields. Looking into the future, we recall that the BW-DCF method relies on separating the intralayer and interlayer contributions in the DCF and the structure factor. Hopefully, this idea might be applied and extended to interpret X-ray and neutron diffraction experiments. The analysis of graphene layers^31^ indicates that a direct link between the theoretical BW analysis and the experimental diffraction data is feasible. Additional work is in progress to account for all the internal fluctuation modes in lipid bilayers, including the extension of the BW treatment to include inter- and intralayer fluctuations.

## Supporting Information Available

Details of numerical simulations, ISM results for MARTINI DPPC–cholesterol and for all-atoms DPPC, analysis of the dependence of the density correlation function and *βκ* with system size, the structure factor of tensionless DPPC–cholesterol and DPPC membranes, and additional results for Figures 5 and 6 not shown in the main text (PDF)
