# Supplementary material for: Bending Modulus of Lipid Membranes from Density Correlation Functions
Source: J Chem Theory Comput. 2022 Apr 7;18(5):3151–63. doi: 10.1021/acs.jctc.2c00099 (PMC9097289; doi:10.1021/acs.jctc.2c00099)
Supplement: Supplementary file 1 — ct2c00099_si_001.pdf [file ct2c00099_si_001.pdf]

Supporting Information (SI)

Bending modulus of lipid membranes from density correlation functions

Jose Hernández-Muñoz,[1] Fernando Bresme,[2] Pedro Tarazona,[1,3] and Enrique Chacón[4]

[1] *Departamento de Física Teórica de la Materia Condensada, IFIMAC Condensed Matter Physics Center, Universidad Autónoma de Madrid, Madrid 28049, Spain*

[2] *Department of Chemistry, Molecular Sciences Research Hub, Imperial College, W12 0BZ, London, United Kingdom*

[3] *Instituto Nicolás Cabrera de Ciencia de Materiales, Universidad Autónoma de Madrid, Madrid 28049, Spain*

[4] *Instituto de Ciencia de Materiales de Madrid, Consejo Superior de Investigaciones Científicas, Madrid 28049, Spain*[a]

(Dated: 21 March 2022)

[a] Electronic mail: echacon@icmm.csic.es

I. SIMULATION PARAMETERS OF THE MEMBRANES ANALYZED IN THIS WORK

In the present work we have analysed simulations of lipid bilayers of 1-palmitoyl-2-oleoyl-sn-glycero-3-phosphocholine (POCP) in the tensionless state and under tension conditions, and 1,2-dipalmitoyl-sn-glycero-3-phosphocholine (DPPC) tensionless bilayers, using the MARTINI[1] MD simulation configurations generated in our previous works[2,3] (see those papers for more details on the simulation approach). For the free POPC[3] we considered systems with different number of lipids, and therefore membranes with different cross sectional areas. These systems were used to analyze the size dependence of our results. The pair interactions were truncated following the MARTINI approach, with a cutoff of 0.9 nm for the Lennard-Jones non-bonding interactions and a 1.2 nm shifted Coulomb potential combined with an effective dielectric constant, which was set to 15, for electrostatic interactions. The systems investigated here are in the L_d phase, or the L_o phase in the DPPC:CHOL system. To control the temperature and pressure we used either the Berendsen thermostat and barostat, or the v-rescale thermostat[4] and the Parrinello-Rahman semi-isotropic barostat, with 2 and 10 ps for the damping constants, respectively. The main advantage of the Parrinello-Rahman barostat is that it reproduces the correct statistical ensemble (surface tension constant) and therefore it is possible to evaluate the area compressibility modulus from the area fluctuations of the membranes[3]. The trajectories were integrated using the Leap-frog algorithm with a timestep of 0.03 ps.

We have performed additional simulation in this work:

- A tensionless DPPC membrane containing cholesterol, at 50:50 (DPPC-Cholesterol) composition, using the MARTINI force-field.
- Tensionless DPPC membranes with the CHARMM36 all-atom force-field.

A. DPPC-Cholesterol 50:50

The characteristics of this new simulation are very similar to the previous simulations of the POPC and the pure DPPC. All the interactions (lipid-lipid, lipid-water and DPPC-cholesterol) were calculated using the MARTINI forcefield, which has been widely used to simulate biological systems, including the phase behavior in the presence of cholesterol[5].

The simulation contained 2304 DPPC and 2304 cholesterol molecules and were performed with the GROMACS simulation package[6].

TABLE I. Simulation parameters of the main systems investigated in this work. Surface tension γ_0, area per phospholipid $a_{\rm Phos}$, box dimensions, $\rm N_{Phos}$ number of phospholipid molecules (DPPC or POPC) per layer, $\rm N_{Water}$ number of water molecules, Δt production simulation time, and $\rm N_{CW}$ number of configurations for the fluctuation analysis . $\langle L_z \rangle \approx 14.0 nm$ for all systems.

Barostat	$\langle L_x \rangle$ (nm)	$\langle L_y \rangle$ (nm)	$a_{\rm Phos}$ (nm^2)	$\rm N_{Phos}$	$\rm N_{Water}$	Δt μs	$\rm N_{CW}$
MARTINI POPC under tension $\gamma_0 = 15.2 mN/m$						$T = 298.15K$	
Berendsen	25.85±0.01	27.19±0.01	0.703±0.001	1000	53000	7.5	20000
Parrinello-Rahman	52.86±0.01	53.17±0.02	0.7027±0.0005	4000	228000	0.274	4569
MARTINI Free POPC $\gamma_0 = 0$						$T = 320K$	
Parrinello-Rahman	12.60±0.01	13.23±0.02	0.6671±0.0002	250	14592	0.450	6001
Parrinello-Rahman	21.83±0.01	22.87±0.02	0.6662±0.0002	750	45000	0.450	6001
Parrinello-Rahman	35.55±0.01	37.42±0.02	0.6653±0.0002	2000	120000	0.450	5934
Parrinello-Rahman	43.54±0.01	45.80±0.02	0.6648±0.0002	3000	100000	0.300	3987
Parrinello-Rahman	50.28±0.01	52.89±0.02	0.6650±0.0002	4000	228000	0.249	3311
MARTINI Free DPPC $\gamma_0 = 0$						$T = 325K$	
Berendsen	25.24±0.01	25.81±0.02	0.636±0.001	1024	53000	7.5	10000
All-Atom Free DPPC $\gamma_0 = 0$						$T = 323.15K$	
Parrinello-Rahman	17.44±0.01	17.44±0.02	0.609±0.001	500	30400	0.3	4000
Parrinello-Rahman	23.41±0.01	23.41±0.02	0.609±0.001	900	54700	0.9	12000
MARTINI Free DPPC-Cholesterol 1:1 $\gamma_0 = 0$						$T = 325K$	
Parrinello-Rahman	30.69±0.01	30.69±0.01	0.818±0.001	1152	53000	0.31	5034

B. All-Atom DPPC

The All-atom simulations of the DPPC bilayers were performed using the CHARMM36 forcefield[7] at 323.15 K. We used the v-rescale thermostat with a time constant of 1 ps, and the Parrinello-Rahman barostat was employed to perform the simulations at constant surface

tension equal to zero, with a pressure normal to the membrane plane of 1 bar. The coupling constant for the barostat was set to 5 ps. We employed the Particle Mesh Ewald method with a cutoff in real space of 1.2 nm, and the Lennard-Jones interactions were truncated at 1.2 nm using a switching function between 1 and 1.2 nm. The initial configurations were generated using CHARMM-GUI[8–10] We performed simulations of bilayer containing 1000 and 1800 DPPC molecules.

In Table I we list the main parameters of the simulations.

II. ISM RESULTS FOR THE DPPC-CHOLESTEROL MEMBRANE

As the DPPC-Cholesterol membrane had not been previously analyzed with the ISM procedure, we include here the ISM results for that case. Figure 1 shows the mean density profile, the intrinsic profile and the mean density profile obtained via the convolution:

$$\rho(z) = \int d\xi \; \mathcal{P}(\xi) \; \rho_{\rm I}(z-\xi), \tag{1}$$

between an *intrinsic density profile*, $\rho_{\rm I}(z)$, and the probability distribution, $\mathcal{P}(\xi)$, of the local values of the intrinsic surface $z = \xi(\vec{x})$.

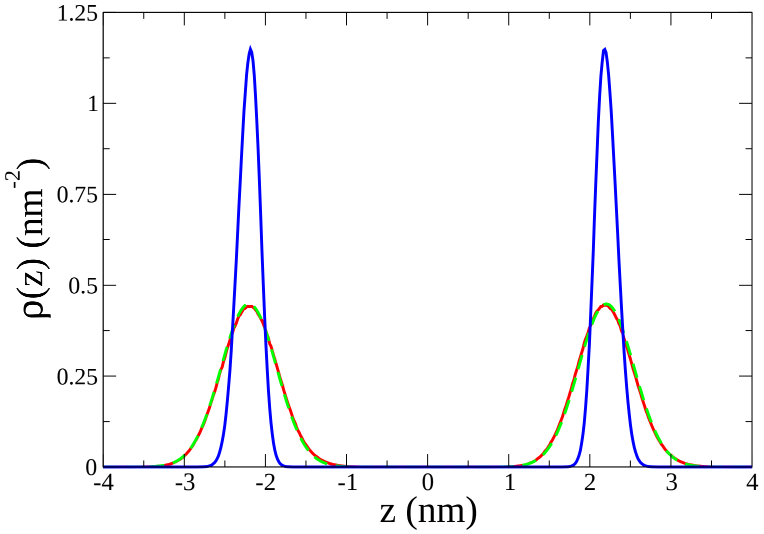

FIG. 1. The DPPC phosphorus bead density profiles of the free DPPC-Cholesterol membrane. Red full line: MD mean density profile $\rho(z)$, within the size ($L_{xy} \approx 51$nm) of our MD box. Blue full line: intrinsic profile $\rho^{in}(z; q_{\rm u})$, independent of L_{xy} and with our cut-off choice $q_{\rm u} = 2.2 \; nm^{-1}$. Dashed green line: mean density profile obtained from the Capillary Wave Theory (eq. 1), with the probability distribution $P(\xi)$ corresponding to the MD-ISM used to get $\rho^{\rm in}(z; q_{\rm u})$.

In Figure 2 we show the surface tension dependence with q for the tensionless DPPC-Cholesterol membrane. In addition to the modes shown in the Figure 1 of the main text we also include the *peristaltic* (P) mode[2], which is given by the local half-thickness $\xi^{\mathrm{P}}(\vec{x}) \equiv [\xi^{+}(\vec{x}) - \xi^{-}(\vec{x})]/2$. The peristaltic mode does not show the divergence $\langle|\hat{\xi}^{\mathrm{x}}_{\vec{q}}|^2\rangle \sim 1/q^2$ characteristic of fluctuating surfaces ($x = m$ or U), therefore it provides a more natural way of defining $u^{\mathrm{P}}(q) = kT/(\langle|\hat{\xi}^{\mathrm{P}}_{\vec{q}}|^2\rangle A_o)$, with a finite limit $u^{\mathrm{P}}(0)$. To obtain a q-dependent surface tension for the P mode, similar to eq (5) of the main text, we define $\gamma^{\mathrm{P}}(q) = u^{\mathrm{P}}(q)/q^2$ and

$$\gamma^{\mathrm{P}}_{\mathrm{eff}}(q) \equiv \frac{k_{\mathrm{B}}T}{q^2\left\langle|\hat{\xi}^{\mathrm{P}}_{q}|^2\right\rangle A_0} \approx \frac{u^{\mathrm{P}}_0}{q^2} + \gamma^{\mathrm{P}}_0 + \kappa^{\mathrm{P}} q^2 \tag{2}$$

which is shown in Figure 2.

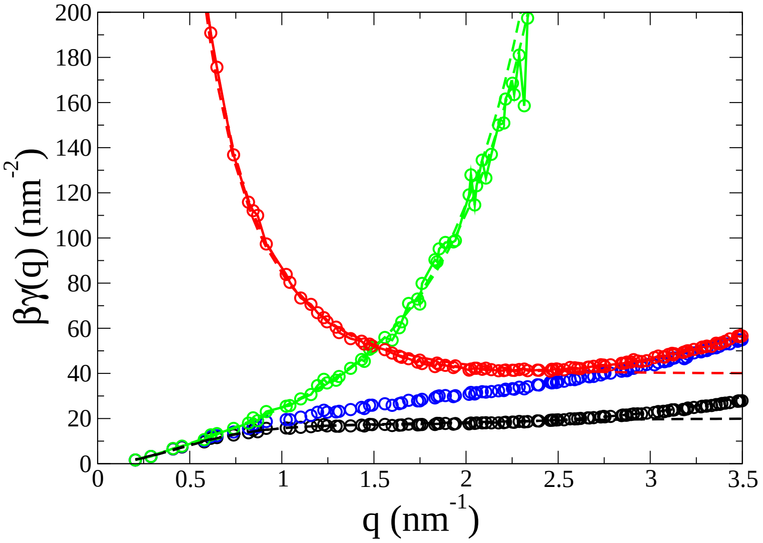

FIG. 2. The surface tensions as function of the wavevector q for the tensionless DPPC-Cholesterol membrane. The circles show our simulation results: green the coupled undulatory mode $\gamma^{\mathrm{CU}}(q)$; red the peristaltic (uncoupled) mode $\gamma^{\mathrm{P}}_{eff}(q)$; blue the undulatory mode $\gamma^{\mathrm{U}}(q)$. and black the monolayer mode $\gamma^{\mathrm{m}}(q)$. The dashed are a guide for the eyes.

As shown in Figure 2 , the addition of cholesterol at (1:1) concentration in DPPC, expands the range of q-vectors ($\gamma^{\mathrm{CU}}(q) < \gamma^{\mathrm{P}}_{eff}(q)$) where the fluctuations of the two lipid layers are correlated.

Of the three modes; U, CU and P, only two are independent and are related to each other by equation[2]:

$$\frac{1}{\gamma^{\mathrm{U}}(q)} = \frac{1}{\gamma^{\mathrm{P}}_{\mathrm{eff}}(q)} + \frac{1}{\gamma^{\mathrm{CU}}(q)}. \tag{3}$$

As explained in the main text, the function $D(q)$ defined by the ratio between the U and CU surface tensions, is expected to behave as

$$D(q) = \frac{\gamma^{\mathrm{U}}(q)}{\gamma^{\mathrm{CU}}(q)} = 1 - \left(\frac{q}{q_d}\right)^4 + \mathcal{O}(q^6). \quad (4)$$

Within of the ISM formalism we can evaluate $D(q)$ directly, and check that indeed it has no q^2 term in its Taylor expansion . In the next figure we show the ISM $D(q)$ for the free DPPC membranes.

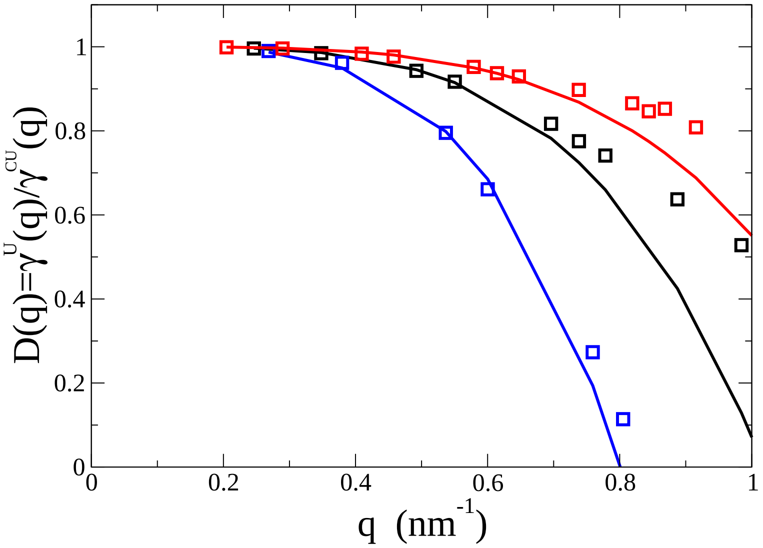

FIG. 3. The symbols show the ISM $D(q) = \gamma^{\mathrm{U}}(q)/\gamma^{\mathrm{CU}}(q)$ function for the tensionless DPPC membranes analyzed in this work. Black: MARTINI DPPC with N=2048, red: MARTINI DPPC-Cholesterol with N=2304, and blue: CHARMM36 All-Atom simulations of DPPC with N=1800 (blue). The lines are the fit to $S(q) = 1 - (q/q_d)^4$ at low q

As we can see to low q all membranes have behavior $S(q) = 1 - (q/q_d)^4$ describe by eq. (4), eq. (19) of the main text. However to high q the behavior of the MARTINI membranes is very different to the All-Atom ones. The All-Atom $D(q)$ quickly changes to negative values while the MARTINI membranes exhibit a positive monotonous decay. Our BW-DFT formalism not have access to $D(q)$ so in eq. (19) of the main text we have used a parametrization (eq. (19) of the main text) which is valid for any forcefield although this reduces the range of q in which the formalism can be used.

Figure 4 presents the simulated density-density correlation functions of DPPC-Cholesterol for three wavevector values. We compare it with the Bedeaux-Weeks prediction $G_{BW}(z_1, z_2; q)$,

using $n = 20$ to calculate the series, and the ISM undulatory $\gamma^{\mathrm{U}}(q)$ (see eq. (6) of the main text).

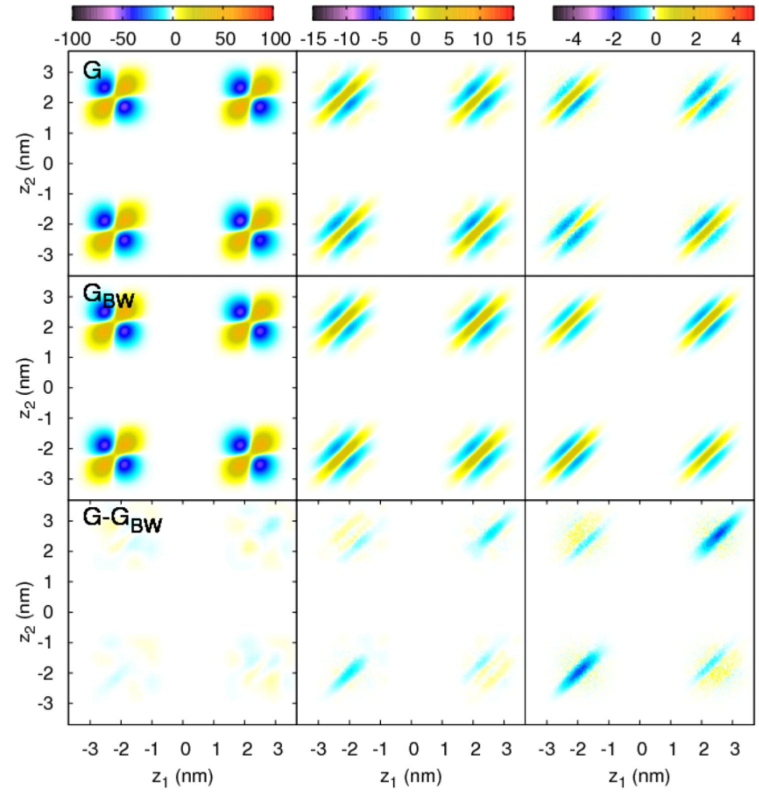

FIG. 4. The density correlation function of DPPC-Cholesterol is presented for three wavevector values. Left column $q = 0.205$ nm^{-1}, middle column $q = 0.614$ nm^{-1}, right column $q = 1.023$ nm^{-1}. Bottom row: the direct MD results $G(z_1, z_2; q)$. Second row: the Bedeaux Weeks theoretical prediction $G_{BW}(z_1, z_2; q)$ to order $n = 20$, using the undulatory mode $\gamma^{\mathrm{U}}(q)$. Third row: $\Delta G = G - G_{\mathrm{BW}}$.

III. ISM RESULTS FOR THE ALL-ATOMS TENSIONLESS DPPC

Figure 5 shows the surface tension dependence with q for the all-atom tensionless DPPC membranes.

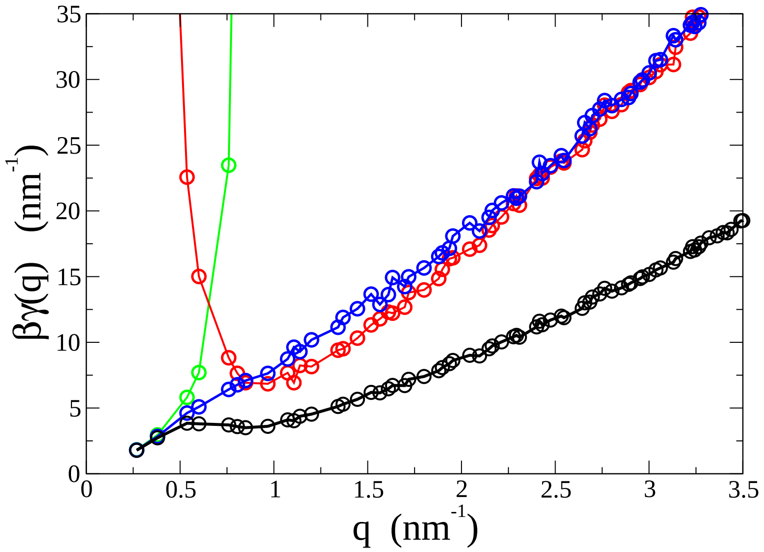

FIG. 5. The surface tensions as function of the wavevector q for the tensionless DPPC with $N = 1000$. The circles show our simulation results: green the coupled undulatory mode $\gamma^{\rm CU}(q)$; red the peristaltic (uncoupled) mode $\gamma^{\rm P}_{eff}(q)$; blue the undulatory mode $\gamma^{\rm U}(q)$. and black the monolayer mode $\gamma^{\rm m}(q)$. The lines are a guide for the eyes.

Compared with the MARTINI forcefield model, the all-atom forcefield for DPPC gives a much narrower window ($\gamma^{\rm CU}(q) < \gamma^{\rm P}_{eff}(q)$) of q values where both lipid layers fluctuate together.

IV. MEAN DENSITY PROFILES AND DENSITY CORRELATION FUNCTIONS OF LARGE MEMBRANES

As shown in Figure 6, for large lateral sizes the mean density profile of the tensionless POPC membrane features two wide peaks that spread over a larger z region, overlapping with each other.

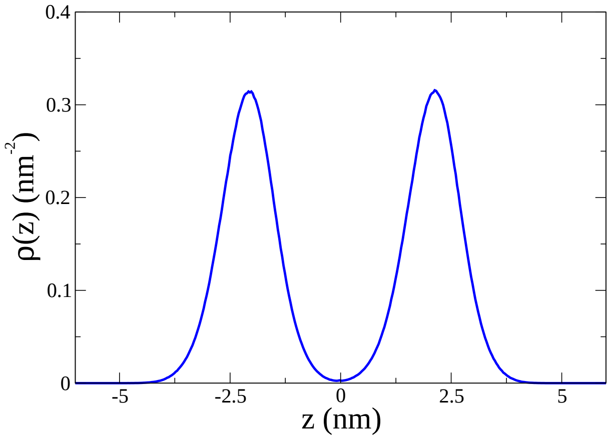

FIG. 6. Simulated mean density profile of the tensionless POPC membrane, obtained using the coordinates of the phosphorous atoms in the lipid head group. The membrane consists of 4000 lipids per layer.

The impact of the larger fluctuations in these large membranes can also be observed in the density correlation functions, with the four quadrants spreading over larger (z_1,z_2) regions and overlapping with each other (see Figure 7) . We have used the same scale for the axes (x,y) as in Figure 2 in the main article to facilitate comparison.

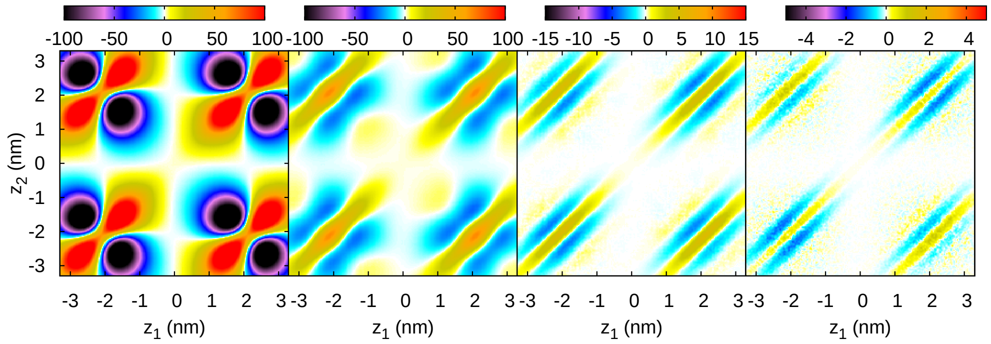

FIG. 7. Direct MD density correlation function of a tensionless POPC membrane with 4000 lipids per layer. for four wavevector values. Left panel $q = 0.122$ nm^{-1}, second panel $q = 0.244$ nm^{-1}, third panel $q = 0.732$ nm^{-1}. nd right panel $q = 1.22$ nm^{-1}.

We show in Figure 8 the impact of the surface tension. Upon applying a tension on the membrane the density correlation shows again four separate quadrants.

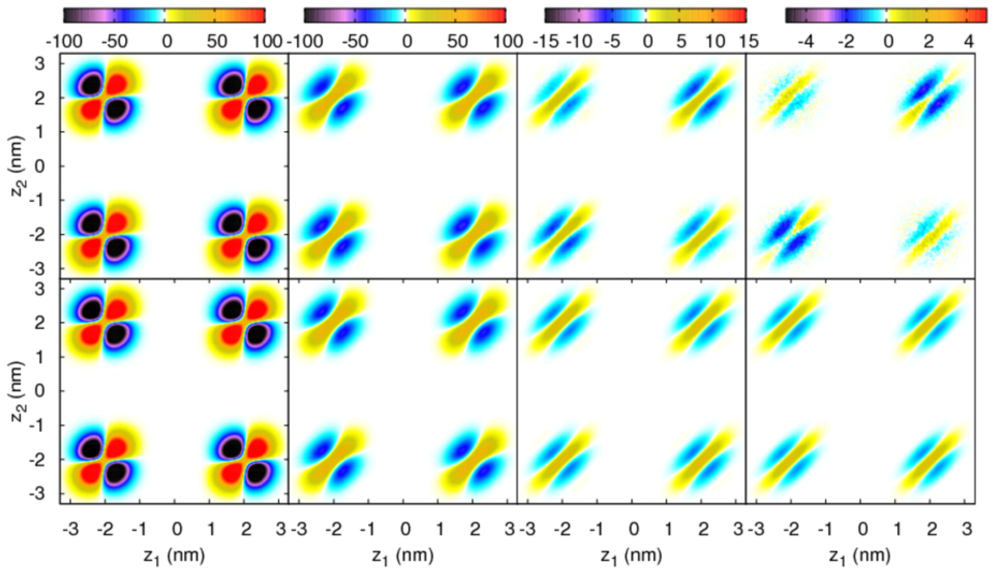

FIG. 8. The density correlation function of POPC under tension ($15.2mN/m$) membrane with 4000 lipids per layer for four wavevector values; left column $q = 0.118$ nm^{-1}, second column $q = 0.237$ nm^{-1}, third column $q = 0.711$ nm^{-1}. and right column $q = 1.18$ nm^{-1}. Top row: the direct MD simulations results, bottom row: the Bedeaux-Weeks (BW) theoretical prediction using the undulatory mode (U) $\gamma^{\mathrm{U}}(q)$.

Notice that the second column of Figure 8 for the membrane with 4000 POPC molecules per layer corresponds to the same $q = 0.118\text{nm}^{-1}$ as the first column of Figure 2 of the main article, for a membrane with 1000 POPC molecules under the same tension. Wertheim's term ($n = 1$ in the BW series) gives the same (size independent) prediction for both systems, but the MD results for $G(z, z', q)$ are clearly different and the full BW series describes well the influence membrane size on the DCF for a given q.

V. THE STRUCTURE FACTOR

In Figure 9 we compare the simulated structure factor $S(q, q_z)$ with the BW structure factor $S_{BW}(q, q_z)$ for a POPC membrane under tension, with 1000 lipids per layer, at the lowest $q = 2\pi/L_x$ compatible with the simulation box.

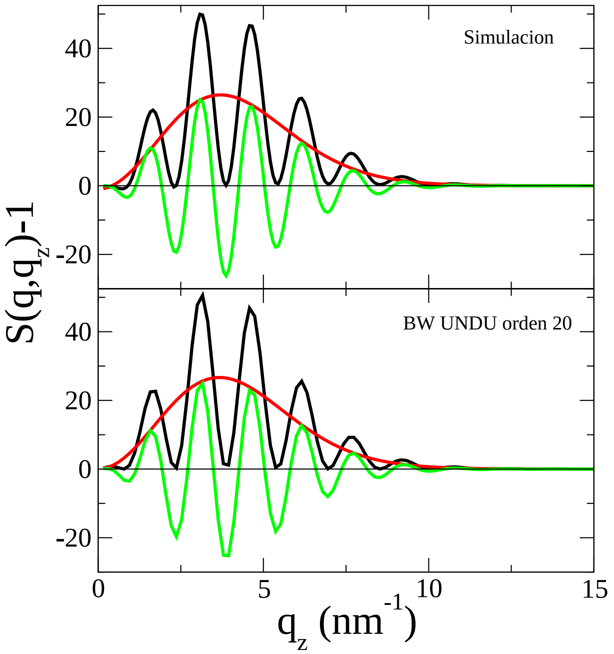

FIG. 9. The interlayer structure factor $S(q, q_z)$ of the POPC membranes under tension at the lowest wavevector $q = 0.237nm^{-1}$ in the MD box with 2000 lipids per layer. Top panel: direct MD results. Bottom panel: Bedeaux-Weeks (BW) theoretical prediction to $n_{\rm BW} = 20$ (see eqs 6 and 9 of the main article), using the undulatory mode (U) $\gamma^{\rm U}(q)$. Black line: total structure factor, blue line: interlayer component and red line: intralayer component.

As we can see at this low wavevector, $q = 2\pi/L_x = 0.237nm^{-1}$, the BW $S_{BW}(q, q_z)$ represents more accurately the simulated $S(q, q_z)$ results than at $q = 10\pi/L_x = 1.18nm^{-1}$ shown in Figure 3 of the main text.

In Figure 10 we compare the interlayer simulation structure factor $S^{+-}(q, q_z)$ with the interlayer BW structure factor $S^{+-}_{BW}(q, q_z)$ to $q = 10\pi/L_x$ for the DPPC-Cholesterol (top panel) and for the tensionless DPPC membranes (bottom panel). This figure is equivalent to Figure 4 of the main text, but now for the tensionless DPPC and DPPC-Cholesterol membranes.

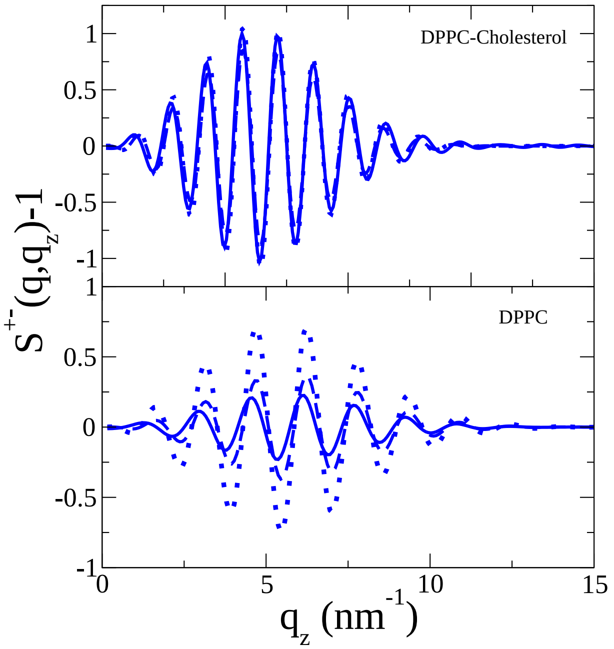

FIG. 10. The interlayer structure factor $S^{+-}(q, q_z)$ with $q = 5\frac{2\pi}{L_x}$ for tensionless DPPC-Cholesterol (top panel) and pure DPPC membranes (bottom panel). The MD results (full lines) are compared with BW predictions $S^{+-}_{\rm BW}(q, q_z)$ (calculated up to $n_{\rm BW} = 20$ order), calculated with $\gamma^{\rm CU}(q)$ (dashed lines) and with $\gamma^{\rm U}(q)$ (dotted lines).

The results for the tensionless DPPC membrane are very similar to those shown in the Figure 4 of the main text for the POPC membrane under tension, and clearly the CU-BW predictions are much closer to the MD results that the U-BW one. The presence of cholesterol maintains the coupling between the lipid layers over a larger interval of wavevectors. Therefore the BW predictions using $\gamma^{\rm U}(q)$ and $\gamma^{\rm CU}(q)$ are much more similar to each other than for a pure DPPC membrane (at the same q values).

VI. BW-DECONSTRUCTION (AT ORDER N=20) FOR THE DPPC AND DPPC-CHOLESTEROL MEMBRANES, TO GET $\gamma^{\rm CU}(q)$ FROM THE INTERLAYER MD $G(z_1, z_2; q)$

We show in Figure 11 a similar plot to that shown in Figure 5 of the main text, but now for tensionless DPPC(MARTINI and All-Atoms) and DPPC-Cholesterol membranes.

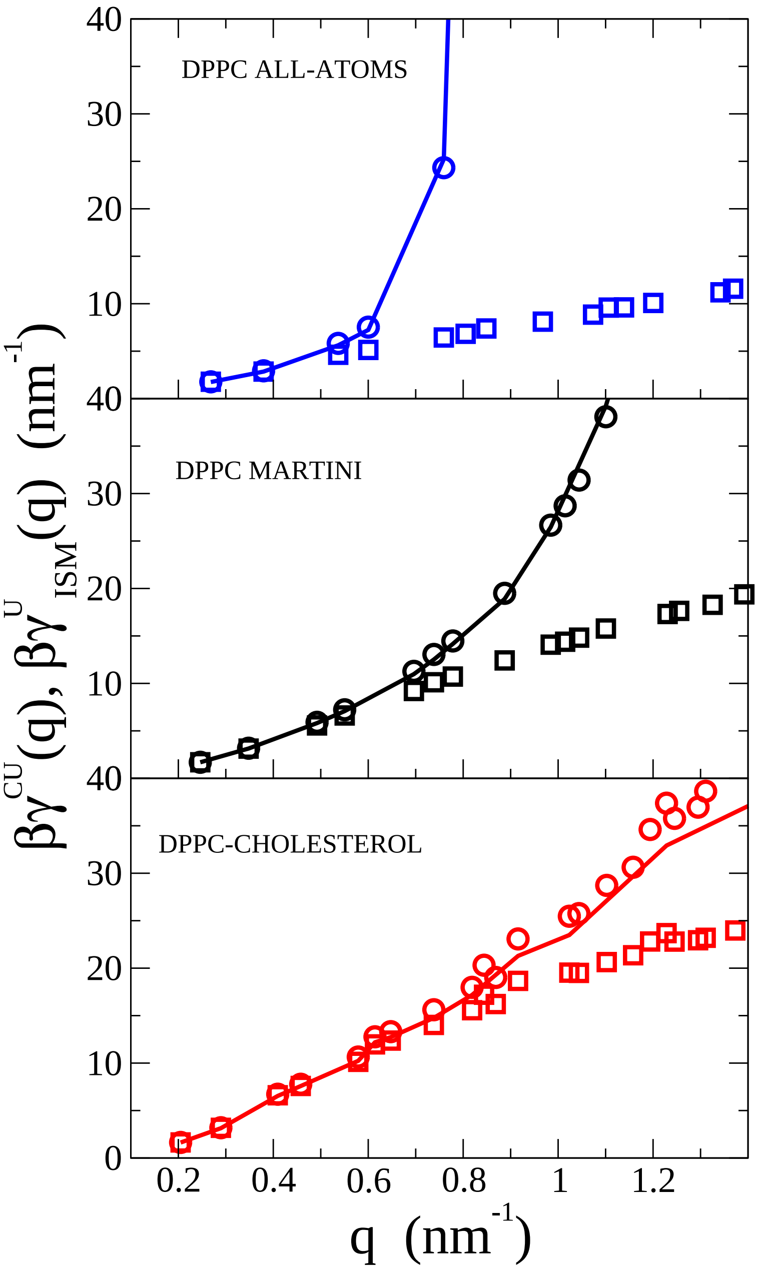

FIG. 11. The wavevector dependent surface tension $\gamma^{\mathrm{CU}}(q)$ for the All-Atoms DPPC (top panel), MARTINI DPPC (middle panel) and MARTINI DPPC-Cholesterol (bottom panel) tensionless membranes. The full line represents the results from the BW-*deconstruction* BW with $n_{BW} = 20$ of MD results for $G_{+-}(z_1, z_2, q)$. The symbols represent the ISM-MD results; circles: $\gamma^{\mathrm{CU}}(q)$, squares: $\gamma^{\mathrm{U}}(q)$.

The Figure 12 is the same Figure 6 of the main text, i.e the inverse of the coupled undulatory surface tension, $\gamma^{\mathrm{CU}}(q)$, for the DPPC membranes analyzed in this work, but

now including the inverse of the usual undulatory surface tension, $\gamma^{\mathrm{U}}(q)$, obtained with the ISM. The $\gamma^{\mathrm{U}}(q)$ function is the usual way to obtain the bending but , as we discuss in the main text, it is not easily obtained from the density correlation function since it contains a large contribution the non-BW background. The BW-DCF route introduced in this work is only applicable to the coupled undulatory mode, $\gamma^{\mathrm{CU}}(q)$.

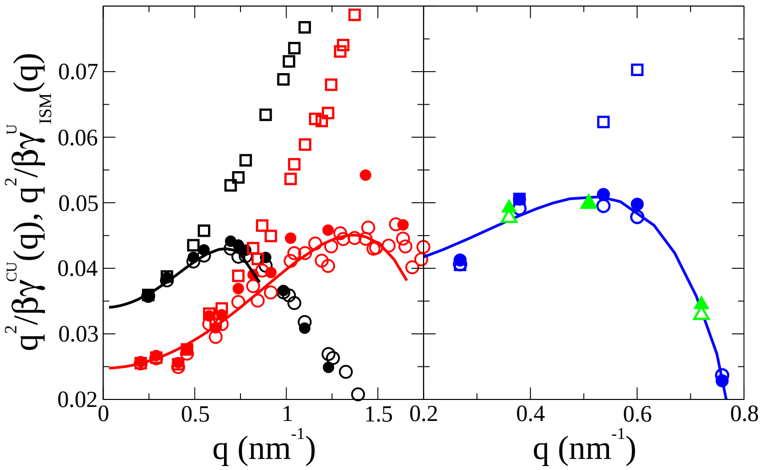

FIG. 12. The inverse of the coupled undulatory surface tension, $\gamma^{\mathrm{CU}}(q)$, for the DPPC membranes analyzed in this work. Left panel: MARTINI tensionless DPPC with N=2048 (black), and MARTINI tensionless DPPC-Cholesterol with N=2304 (red). Right panel: CHARMM36 All-Atom simulations of tensionless DPPC with N=1800 (blue) and N=1000 (green) The empty circles represent the simulation data for the ISM results and the full circles the results from the BW *deconstruction* BW to order $n_{\mathrm{BW}} = 20$ of the simulated data, $G_{+-}(z_1, z_2, q)$. The solid lines show the fittings to the ISM (for the All-Atoms N=1800 of the BW) data to eq. 19 of the main text in the range $0 < q < 0.9q_d$. The empty square symbols are the ISM results for the inverse of the undulatory surface tension, $\gamma^{\mathrm{U}}(q)$.

The next Figure 13 is a similar plot to the previous figure, but now for the POPC membranes free and under tension.

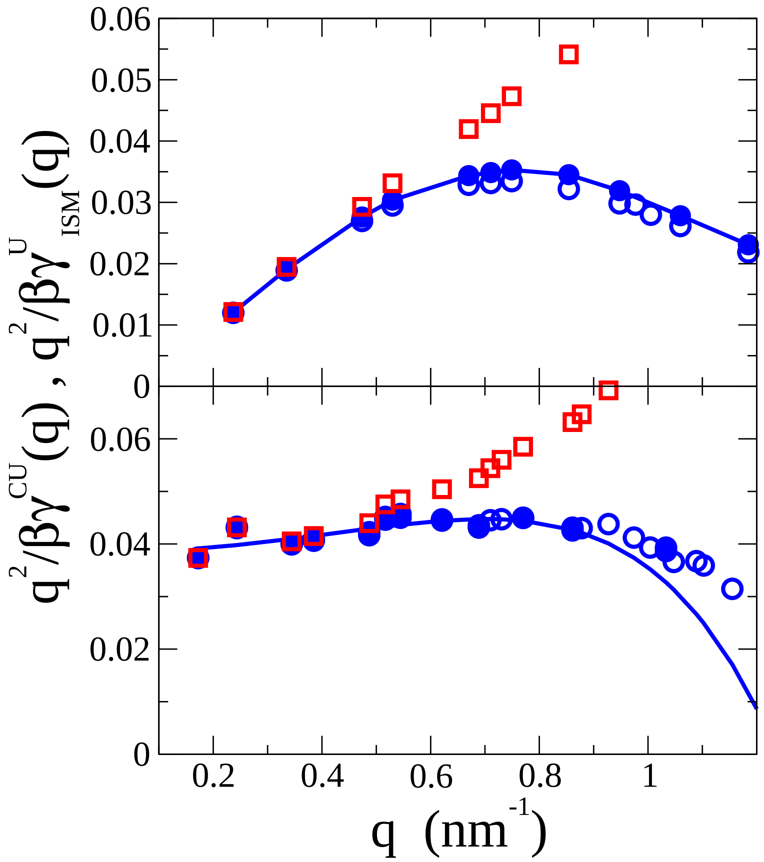

FIG. 13. The inverse of the coupled undulatory surface tension $\gamma^{\mathrm{CU}}(q)$ for the MARTINI force field. Top panel: the POPC under tension N=2000. Bottom panel: free POPC with N=4000. The symbols represent the simulation data; empty circle; the ISM data, and full circle; the *deconstruction* BW to order $n_{BW} = 20$ of $G_{+-}(z_1, z_2, q)$. The solid lines are the fit of the ISM data to eq. 19 of the main text in the range $0 < q < 0.9q_d$ The empty square symbols are the ISM results for the inverse of the undulatory surface tension, $\gamma^{\mathrm{U}}(q)$.

VII. ROBUSTNESS OF THE FITS TO $\frac{q^2}{\gamma^{\mathbf{CU}}(q)}$ FOR THE MARTINI FREE POPC MEMBRANES

In this section we check, for the MARTINI free POPC membranes, the robustness with the lateral size of the membranes. of the fits of $\frac{q^2}{\gamma^{\mathrm{CU}}(q)}$ to the parametric form given by eq. (19) of the main text. For this we have used the simulations MARTINI free POPC membranes carried out in our previous work[3]. As shown in table II the agreement between the BW-DCF and ISM data is good across all the systems studied.

TABLE II. Bending modulus κ and tilt modulus κ_θ for POPC with the coarse grained MARTINI forcefields. N represents the total number of lipids, and $\beta\gamma_0$ the surface tension applied to the membrane. The calculations with the ISM and BW-DCF were performed fittings the coupled-undulatory $\gamma^{\mathrm{CU}}(q)$ mode (eq. (19) of the main text) q_{d} represents the decoupling parameter.

N Model	Method	$\beta\kappa$	κ_θ mN/m	q_{d} nm^{-1}
POPC free				
1500 MARTINI	M_{ISM}	26.49	125	1.026
	M_{BW-DCF}	26.48	108	1.003
4000 MARTINI	M_{ISM}	25.98	191	1.23
	M_{BW-DCF}	25.90	175	1.191
6000 MARTINI	M_{ISM}	25.70	160	1.167
	M_{BW-DCF}	25.73	135	1.138

We note that use of the BW-DCF procedure requires sufficiently large system sizes, typically $N > 1500$. For smaller system sizes the number the points within of the relevant fitting interval $0 < q < q_d$ (where the CWT and the low-q approximation to $D(q)$ (see eq. (4) do not apply) is reduced drastically. However, if we use the ISM values for $D(q)$ instead of the low q approximation we can obtain acceptable estimation for the bending modulus using systems containing $N = 500$ lipids. This analysis falls outside the main objective of this work.

REFERENCES

[1] S. Marrink, H. Risselada, S. Yefimov, D. Tieleman, and A. de Vries, J. Phys. Chem. B **111**, 7812 (2007).

[2] P. Tarazona, E. Chacón, and F. Bresme, J. Chem. Phys. **139**, 094902 (2013).

[3] E. Chacón, P. Tarazona, and F. Bresme, J. Chem. Phys. **143**, 034706 (2015).

[4] G. Bussi, D. Donadio, and M. Parrinello, The Journal of Chemical Physics **126**, 014101 (2007), https://doi.org/10.1063/1.2408420.

[5] Y. Zhang, A. Lervik, J. Seddon, and F. Bresme, Chemistry and physics of lipids **185**, 88 (2015).

[6] B. Hess, C. Kutzner, D. van der Spoel, and E. Lindahl, J. Chem. Theory Comput. **4**, 435 (2008).

[7] J. Klauda, R. Venable, J. Freites, J. O'Connor, D. Tobias, C. Mondragon-Ramirez, I. Vorobyov, A. M. Jr, and R. Pastor, J. Phys. Chem. B **114**, 7830 (2010).

[8] S. Jo, T. Kim, V. G. Iyer, and W. Im, Journal of Computational Chemistry **29**, 1859 (2008), https://onlinelibrary.wiley.com/doi/pdf/10.1002/jcc.20945.

[9] B. R. Brooks, C. L. Brooks III, A. D. Mackerell Jr., L. Nilsson, R. J. Petrella, B. Roux, Y. Won, G. Archontis, C. Bartels, S. Boresch, A. Caflisch, L. Caves, Q. Cui, A. R. Dinner, M. Feig, S. Fischer, J. Gao, M. Hodoscek, W. Im, K. Kuczera, T. Lazaridis, J. Ma, V. Ovchinnikov, E. Paci, R. W. Pastor, C. B. Post, J. Z. Pu, M. Schaefer, B. Tidor, R. M. Venable, H. L. Woodcock, X. Wu, W. Yang, D. M. York, and M. Karplus, Journal of Computational Chemistry **30**, 1545 (2009), https://onlinelibrary.wiley.com/doi/pdf/10.1002/jcc.21287.

[10] J. Lee, X. Cheng, J. M. Swails, M. S. Yeom, P. K. Eastman, J. A. Lemkul, S. Wei, J. Buckner, J. C. Jeong, Y. Qi, S. Jo, V. S. Pande, D. A. Case, C. L. Brooks, A. D. MacKerell, J. B. Klauda, and W. Im, Journal of Chemical Theory and Computation **12**, 405 (2016).
